# Th1 and Th2 cells in equine endometrosis and their interactions with endometrial fibroblasts

**DOI:** 10.1038/s41598-025-20152-0

**Published:** 2025-10-16

**Authors:** Anna Wójtowicz, Agnieszka Sadowska, Tomasz Molcan, Magda Słyszewska, Ewa Monika Drzewiecka, Dawid Tobolski, Graça Ferreira-Dias, Anna Szóstek-Mioduchowska

**Affiliations:** 1 Institute of Animal Reproduction and Food Research of Polish Academy of Sciences, Tuwima 10, 10-748 Olsztyn, Poland; 2 Molecular Biology Laboratory, Polish Academy of Sciences, Institute of Animal Reproduction and Food Research, Tuwima 10, 10-748 Olsztyn, Poland; 3 Department of Large Animal Diseases and Clinic, Institute of Veterinary Medicine, Warsaw University of Life Sciences, 02-787 Warsaw, Poland; 4 Faculty of Veterinary Medicine, CIISA—Center for Interdisciplinary Research in Animal Health, University of Lisbon, Lisbon, Portugal; 5 Associate Laboratory for Animal and Veterinary Sciences, AL4AnimalS, Lisbon, Portugal

**Keywords:** Th1, Th2, Fibrosis, Fibroblasts, Endometrium, Mare, Adaptive immunity, Cytokines, Reproductive disorders, Bioinformatics, Gene expression analysis, Genomic analysis, Sequencing

## Abstract

Mare endometrosis is a chronic degenerative condition of the endometrium, primarily characterized by fibrosis, involving interactions among fibroblasts, immune cells, and epithelial cells regulated by cytokines and growth factors. T helper (Th)1 and Th2 cells seem to play a pivotal role in fibrosis. However, their roles in equine endometrial fibrosis remain unknown. This study explores Th1 and Th2 cell distribution across different stages of endometrium histopathological Kenney and Doig categories; and evaluated their secretome effects on non-fibrotic endometrium derived fibroblast functional characteristics, extracellular matrix (ECM)-associated mRNA transcription, and transcriptomic profiles. Th1 and Th2 cells, along with cytokines (IFN-γ, IL-4, IL-13) and their receptors, were present in mare endometria at all endometrium stages. Th1 secretome influenced genes enriched in metabolism, cell cycle, and ECM-related pathways, while Th2 secretome regulated genes enriched in tissue remodeling and signaling pathways, suggesting their role in the development of fibrosis in the endometrosis progression.

**Supplementary Information:**

The online version contains supplementary material available at 10.1038/s41598-025-20152-0.

## Introduction

Mare endometrosis is a chronic, degenerative condition of the endometrium, characterized mainly by the progressive accumulation of extracellular matrix (ECM) components within the endometrial stroma accompanied by disruption of glandular structures^1,2^. It is important to distinguish between the terms endometrosis and endometriosis, as they describe different conditions and should not be used interchangeably. Endometriosis refers to the extrauterine implantation of endometrial tissue, a condition specific to women^3^. In contrast, endometrosis pertains to degenerative changes in the mare’s endometrium, historically referred to as chronic degenerative endometritis^4^.

Alterations of endometrium accompanying endometrosis compromise the functional capacity of the endometrium, thereby contributing to infertility in mares, particularly as they age. This condition is also associated with inflammatory infiltrates, dilation of endometrial glands, and lymphatic vessels within the equine endometrium ^1,2,5^. Persistent inflammation, disrupted immune responses, and reduced immune tolerance further contribute to the development of endometrosis. Early signs include morphological and functional abnormalities in periglandular stromal cells ^6^. As the condition advances, the tissue shifts to increased populations of fibroblasts and myofibroblasts, either metabolically active or inactive, driving fibrosis progression ^6–8^. Despite years of research on endometrosis, the pathogenesis of this condition remains incompletely understood, which has resulted in a lack of satisfactory treatment options ^4,9^. The results of the recent study indicated an alteration in the expression of genes associated with inflammation, cellular infiltration of macrophages, neutrophil extracellular trap signaling pathway and T helper (Th)1 and Th2 activation pathways in the mare endometrium at the different stages of endometrosis ^10^. Th1 cells are primarily associated with pro-inflammatory responses, producing cytokines such as IFN-γ that activate macrophages and promote cellular immunity ^11,12^. In contrast, Th2 cells are involved in pro-resolving and anti-inflammatory pathways, secreting cytokines like IL-4 and IL-10 that support humoral immunity and tissue repair ^11,13^. These findings suggest that pro-inflammatory cytokines and growth factors, by recruiting inflammatory cells perpetuating chronic inflammation and contributing to fibrotic tissue remodeling ^14^ may play a crucial role in the development of mare endometrosis.

It is well established that Th1 and Th2 cells are considered to be critical components of the immune response. However, a growing body of evidence from studies on animal models of fibrotic disorders suggests roles for Th cell in the development of tissue fibrosis. The role of Th1 and Th2 cell subsets in the development of fibrosis in various organs and tissues is a topic of ongoing research, as previously reviewed ^15^. In a murine model of radiation-induced lung fibrosis, the infiltration of Th cells was observed, indicating their importance in the development of fibrosis ^16^. Results of the study on patients with progressive fibrosing interstitial lung diseases demonstrated increased numbers of Th2 cells in both peripheral blood and bronchoalveolar lavage fluid^17^. In patients with renal fibrosis and in mice with experimentally induced renal fibrosis, elevated numbers of Th2 cells have been observed. Moreover, the Th2 to Th1 cell ratio has been shown to increase in parallel with the development of experimentally induced renal fibrosis in mice^18^. The results of studies analyzing the role of Th1 and Th2 cells in the development of fibrosis indicate that Th1 cells may play an anti-fibrotic role, while Th2 cells may have a profibrotic effect^15,19^. It has been demonstrated that cytokines secreted by Th cells exert an influence on fibroblast activity, which produce and remodel the ECM^20^. More recent studies indicate that cytokines secreted by Th1 cells, such as interferon (IFN)-γ, can modulate inflammation and processes associated with the development of fibrosis^21^. In turn, Th2 cells secrete cytokines, including interleukin (IL)-4 and IL-13, mount type 2 innate responses and modulate tissue repair by affecting fibroblast activity, collagen (COL) production and tissue remodeling^22^. Nevertheless, interpreting the results requires caution, as Th cell subpopulations in fibrotic diseases and experimental animal models may either arise from fibrosis progression or from the accompanying inflammatory response^23,24^. Nevertheless, the distribution and potential role of Th1 and Th2 cell subsets in the pathogenesis of fibrosis in the progression of endometrosis remains to be elucidated.

Some findings suggest that immune cells, beyond their traditional roles in host defense, actively influence non-immune cells, such as fibroblasts and epithelial cells, thereby modulating their functions and contribute to broader physiological and pathological processes^25,26^. The interplay between Th cells and fibroblasts is mediated through cytokine signaling, where Th cells secrete factors that influence fibroblast proliferation, differentiation, and ECM production. This interaction plays a pivotal role in processes such as tissue repair, remodeling, and the progression of fibrosis^27^. The equilibrium between distinct T cell subsets has been demonstrated to influence the degree of fibrosis^28^. Moreover, our previous study indicated changes in expression of genes involved in immune resonse, IL-13 signaling pathway as well as Th1 and Th2 cell differentiation pathways in mare endometria along with the development of endometrosis^10^. Thus, a comprehensive understanding of the role of Th1 and Th2 cells in the development of endometrial fibrosis in the progression of mare endometrosis will indicate the path for further research into the mechanisms of endometrosis development. Therefore, we aimed to determine the distribution of Th1 and Th2 cells in mare from healthy endometrium to endometrosis establishement, and to investigate the influence of Th1 and Th2 cell secretome on mare endometrial fibroblast functional characteristics (proliferation and viability), transcriptomic changes, as well as the mRNA transcription of selected ECM-associated markers.

## Results

**Fig. 1:**
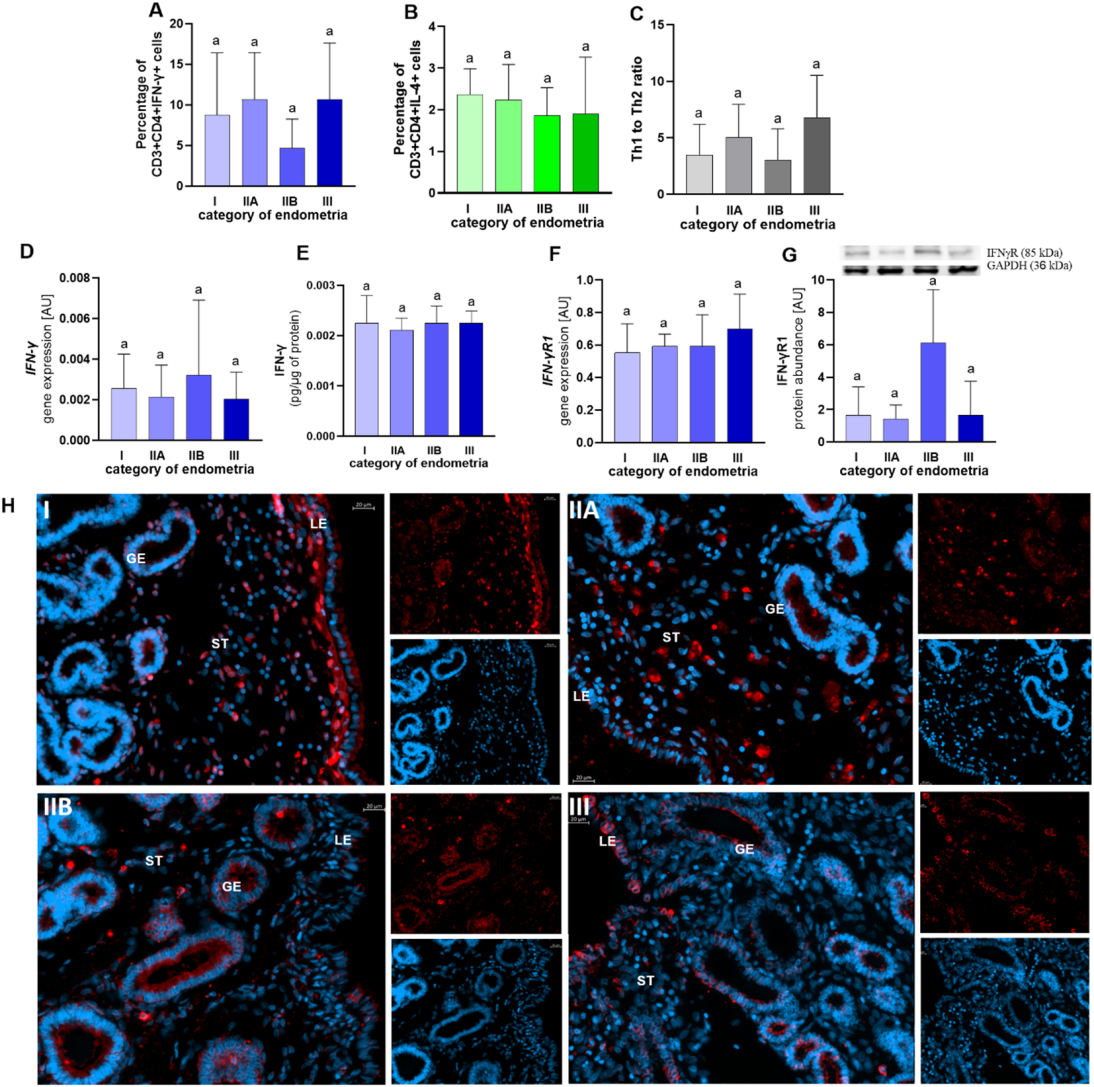
The percentage of Th1 cells (CD3^+^CD4^+^IFN-γ^+^; **A**) and Th 2 cells (CD3^+^CD4^+^IL-4^+^; **B**) as well as the ratio of Th1 cells (CD3^+^CD4^+^IFN^+^) to Th 2 cells (CD3^+^CD4^+^IL-4^+^; **C**) in categories I (no fibrosis), IIA (mild fibrosis), IIB (moderate fibrosis), and III (severe fibrosis) endometria according to Kenney and Doig. The mRNA transcription and concentration of interferon (IFN)-γ (**D**, **E**) and the mRNA transcription and protein abundance of IFN-γ receptor (IFN-γR1; **F**, **G**) in categories I, IIA, IIB, and III endometria of mares. Results are presented as a mean ± standard deviation (SD). Data were analysed using a one-way ANOVA test (p > 0.05). The localization of IFN-γ (H) in categories I, IIA, IIB, and III endometria of mares. LE—luminal epithelium, GE—glandular epithelium, ST—stroma. The bar = 50 µm.

**Fig. 2:**
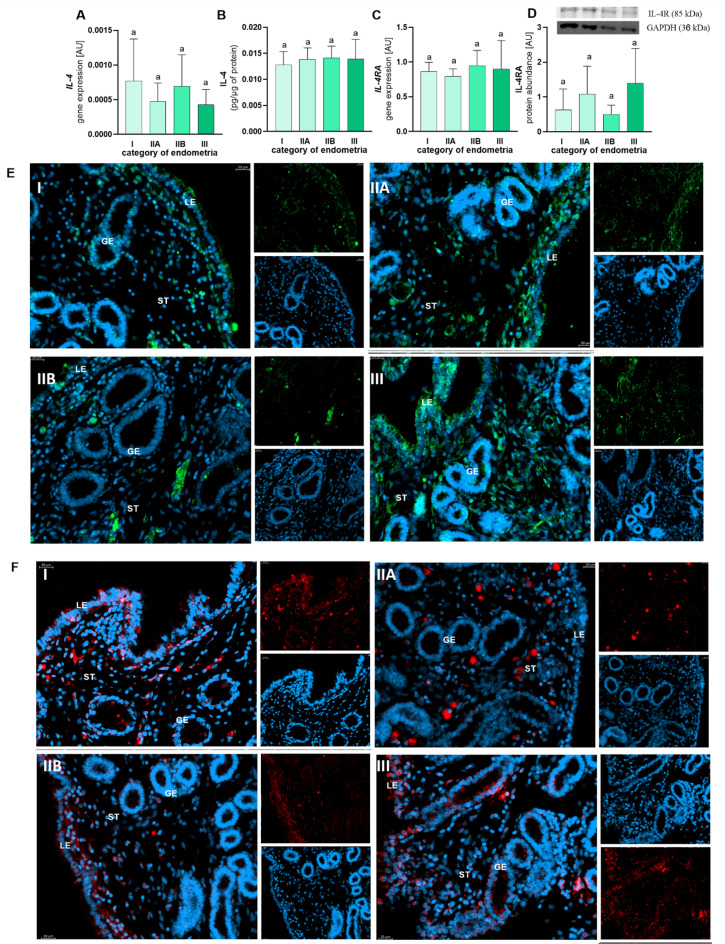
The gene expression and concentration of interleukin (IL)-4 (**A**, **B**) and the gene expression and protein abundance of IL-4 receptor (IL-4RA; **C**, **D**) in categories I (no fibrosis), IIA (mild fibrosis), IIB (moderate fibrosis), and III (severe fibrosis)  endometria according to Kenney and Doig. Results are presented as a mean ± standard deviation (SD). Data were analysed using a one-way ANOVA test (p > 0.05). The localization of IL-4 (**E**) and IL-4RA (**F**) in categories I, IIA, IIB, and III endometria of mares. LE—luminal epithelium, GE—glandular epithelium, ST—stroma. E, F: DAPI—nucleus, blue; green—IL-4; red—IL-4R.

**Fig. 3:**
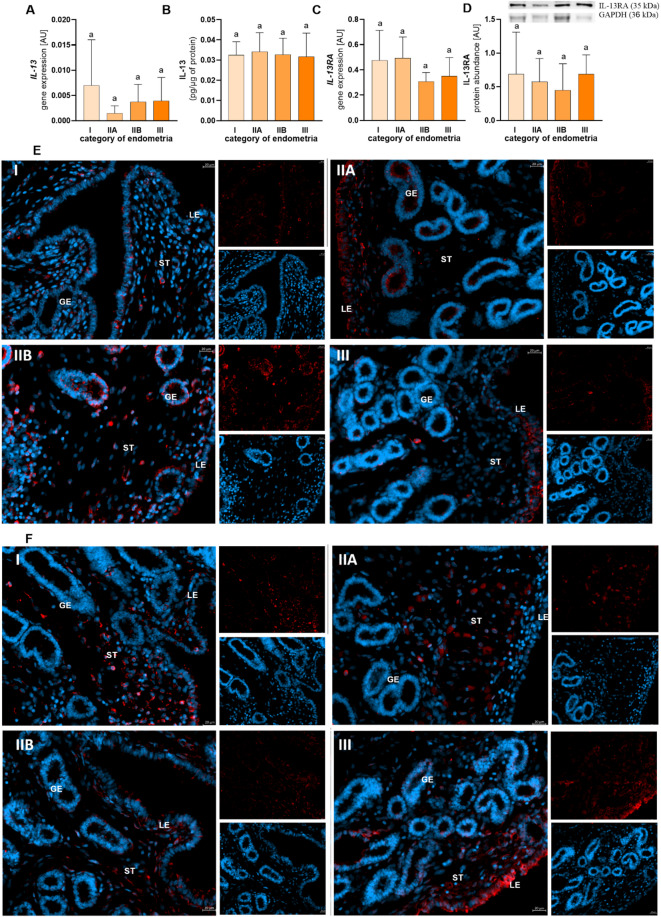
The mRNA transcription and concentration of interleukin (IL)-13 (**A**, **B**) and the mRNA transcription and protein abundance of IL-13 receptor (IL-13A; **C**, **D**) in categories I (no fibrosis), IIA (mild fibrosis), IIB (moderate fibrosis), and III (severe fibrosis) endometria according to Kenney and Doig. Results are presented as a mean ± standard deviation (SD). Data were analysed using a one-way ANOVA test (p > 0.05). The localization of IL-13 (**E**) and IL-13RA (**F**) in categories I, IIA, IIB, and III endometria of mares. LE—luminal epithelium, GE—glandular epithelium, ST—stroma. E, F: DAPI—nucleus, blue; red—IL-13, IL-13R.

The presence of Th1 and Th2 cells in the endometrium of mares with endometrosis was confirmed. No alterations were observed in the distribution of Th1 (Fig. 1A) or Th2 (Fig. 1B) and Th1 to Th2 ratio (Fig. 1C) in the endometrium, between categories of mare endometrosis (p > 0.05). Furthermore, there were no alterations in IFN-γ, IL-4, and IL-13 mRNA transcrition and concentration, as well as mRNA transcription and protein abundance in their receptors (IFN-γR1, IL-4RA, and IL-13RA) between endometrial categories follicular phase of the oestrous cycle (Fig. 1–3; p > 0.05). The localization of IFN-γ, IL-4, IL-4RA, IL-13, and IL-13RA is depicted in the Figs. 1H, 2E,F, and 3E,F.

**Fig. 4:**
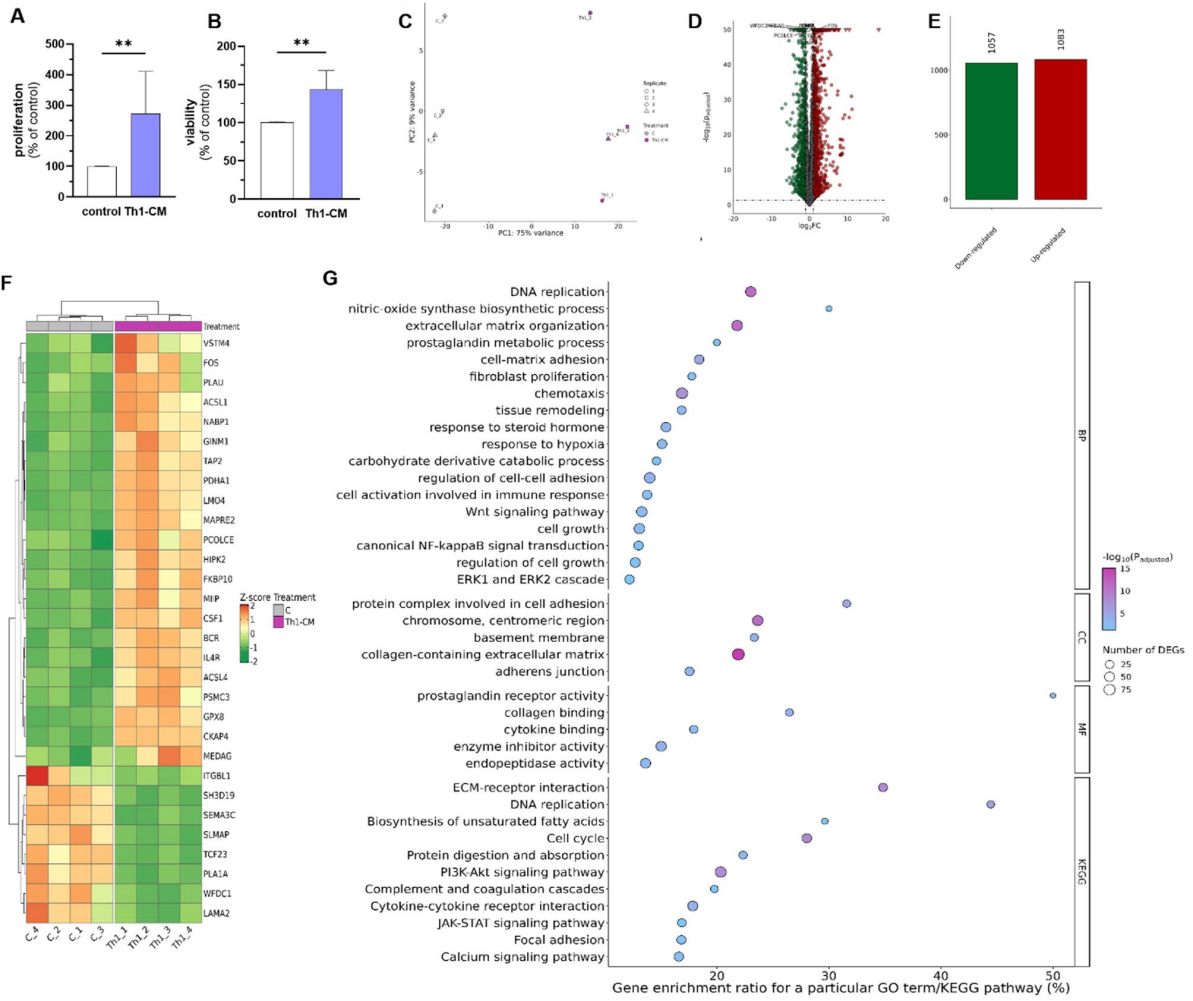
The effect of Th1-conditioned medium (Th1-CM) treatment on proliferation (**A**) and viability (**B**) of mare endometrial fibroblasts after 48 h. Results were calculated as the mean ± standard deviation (SD) and expressed as a percentage of the respective control group. Asterisks denote statistical differences (** p < 0.01). The differences were determined using the unpaired Student-t test. Graphical presentation of the first (PC1) and second (PC2) principal components (PCA) of the mRNA expression pattern in mare endometrial fibroblasts treated with Th1-CM (**C**). The volcano plot (**D**) presents differentially expressed genes (DEGs; padjusted < 0.05 and log2FC ≥ 1.0 or log2FC ≤ −1.0) which are represented by multi-coloured circles, where red colour is up-regulated genes and green down-regulated genes. (**E**) The number of identified up- and down-regulated DEGs. Heatmap (**F**) illustrates the expression profile of top 30 DEGs, selected by lowest padjusted values. The colour scale of the heatmap shows the normalized (Z-score) expression level where red blocks represent up-regulated DEGs, and green blocks represent down-regulated DEGs. C—control group (untreated cells). Selected Gene Ontology (GO) and Kyoto Encyclopedia of Genes and Genomes (KEGG) terms enriched by identified DEGs (padjusted < 0.05 and log2FC ≥ 1.0 or log2FC ≤ −1.0) in fibroblasts derived from category I endometria (n = 4) treated with Th1-CM for 48 h (**G**). The y-axis is GO/KEGG categories. The x-axis refers to GO/KEGG enrichment. BP–Biological Processes, CC—cellular components, MF—molecular functions.

The treatment of mare endometrial fibroblast with Th1-conditioned medium (Th1-CM) increased fibroblast proliferation and viability, as presented in the Fig. 4A, B. Moreover, Th1-CM treatment induced transcriptomic changes in mare endometrial fibroblasts, as presented in Fig. 4C–F. The PCA results of mRNA expression patterns in fibroblasts derived from category I endometrium in response to Th1-CM treatment are presented in Fig. 4C. The treatment of fibroblasts derived from category I endometria with Th1-CM for 48 h resulted in identification of 2140 DEGs (p_adjusted_ ≤ 0.05 and log2FC ≥ 1.0 or log2FC ≤ -1.0). Out of these, expression of 1057 was down-regulated, while expression of 1083 was up-regulated, as presented in Fig. 4E. The five most upregulated DEGs were *COL6A5, CSF3R, ALOX15, NTRK1*, and *PDCD1LG2.* The five most significantly downregulated genes after Th1-CM treatment were *PRLR, CCDC3, DYSF, LMCD1*, and *ADAMTS8.* The full list of DEGs identified in this research task is provided in the Supplementary Table 1. Differentially expressed genes identified in fibroblasts treated with Th1-CM, derived from category I endometria, were subjected to functional analyses. The analysis involving Gene Ontology (GO) and Kyoto Encyclopedia of Genes and Genomes database (KEGG) indicated 902 biological processes (BP), 70 cellular components (CC), and 93 molecular function (MF) terms, as well as 19 KEGG pathways in which identified DEGs may be involved. Selected most enriched GO terms and KEGG pathways are presented in Fig. 4G, while the full list of GO and KEGG terms in enclosed in the Supplementary Table 2.

**Fig. 5:**
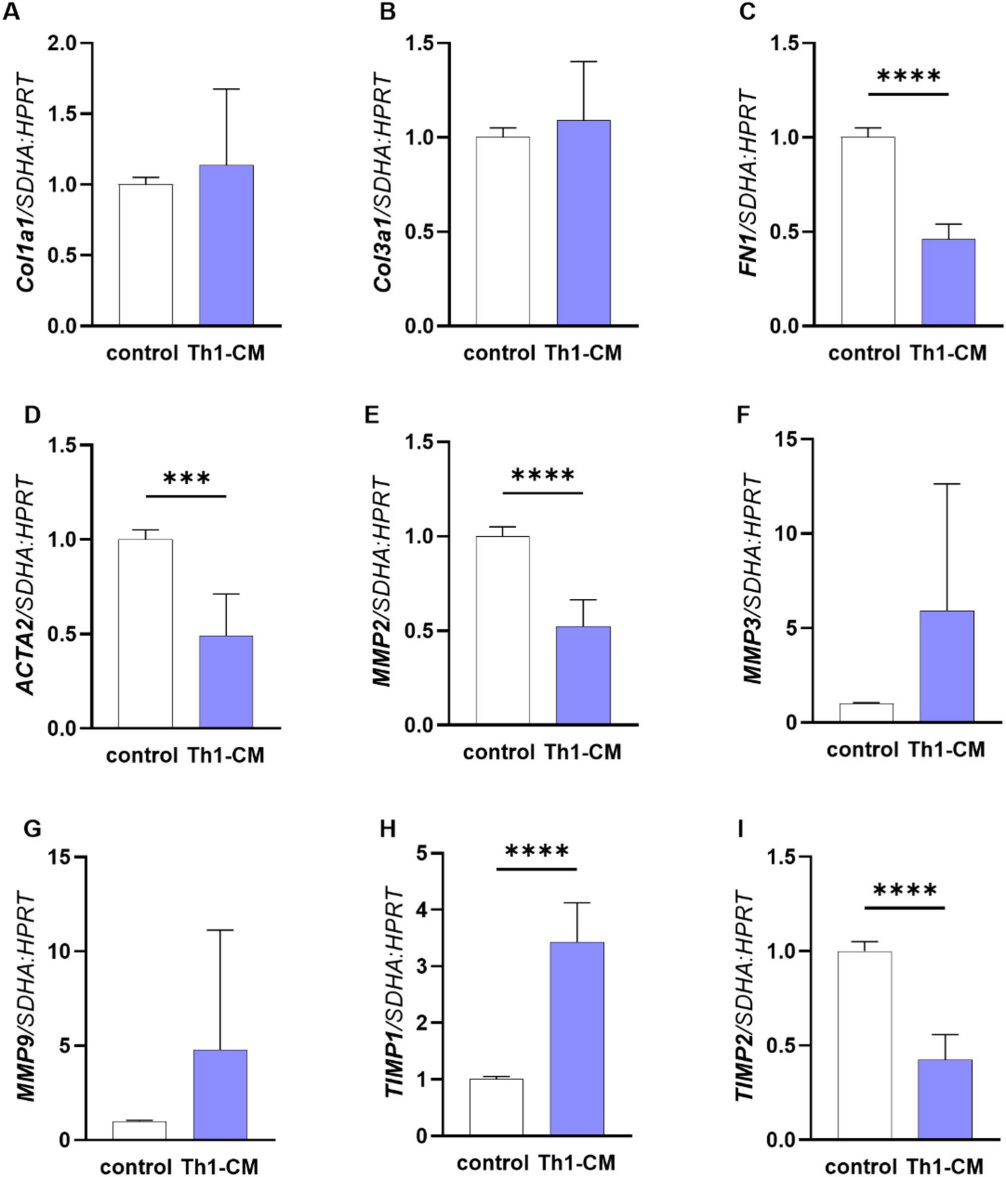
The effect of Th1-conditioned medium (Th1-CM) on mRNA transcription of *collagen (Col)1a1* (**A**), *Col3a1* (**B**), *fibronectin 1* (*FN1;* **C**), *α-smooth muscle actin (ACTA2*; **D**), *matrix metalloproteinase (MMP) 2* (**E**), *MMP3* (F), *MMP9* (**G**), *tissue inhibitor of matrix metalloproteinase* (*TIMP)1* (**H**), and *TIMP2* (**I**) in fibroblasts derived from category I endometria after 48 h of treatment. Results were calculated as the mean ± standard deviation (SD) and expressed as a fold change to the control group. Asterisks denote statistical differences (*** p < 0.001, **** p < 0.0001), as determined by unpaired Student t-test.

The treatment of category I derived fibroblasts with Th1-CM decreased *FN1* (Fig. 5C; p < 0.0001), *ACTA2* (Fig. 5D; p < 0.001), *MMP2* (Fig. 5E; p < 0.0001), *TIMP2* (Fig. 5I; p < 0.0001) and increased *TIMP1* (Fig. 5H; p < 0.0001) mRNA transcription, after 48 h. No changes of *Col1a1* (Fig. 5A)*, Col3a1* (Fig. 5B)*, MMP3* (Fig. 5F)*,* and *MMP9* (Fig. 5G) mRNA expression were observed in fibroblasts treated with Th1-CM (p > 0.05).

**Fig. 6:**
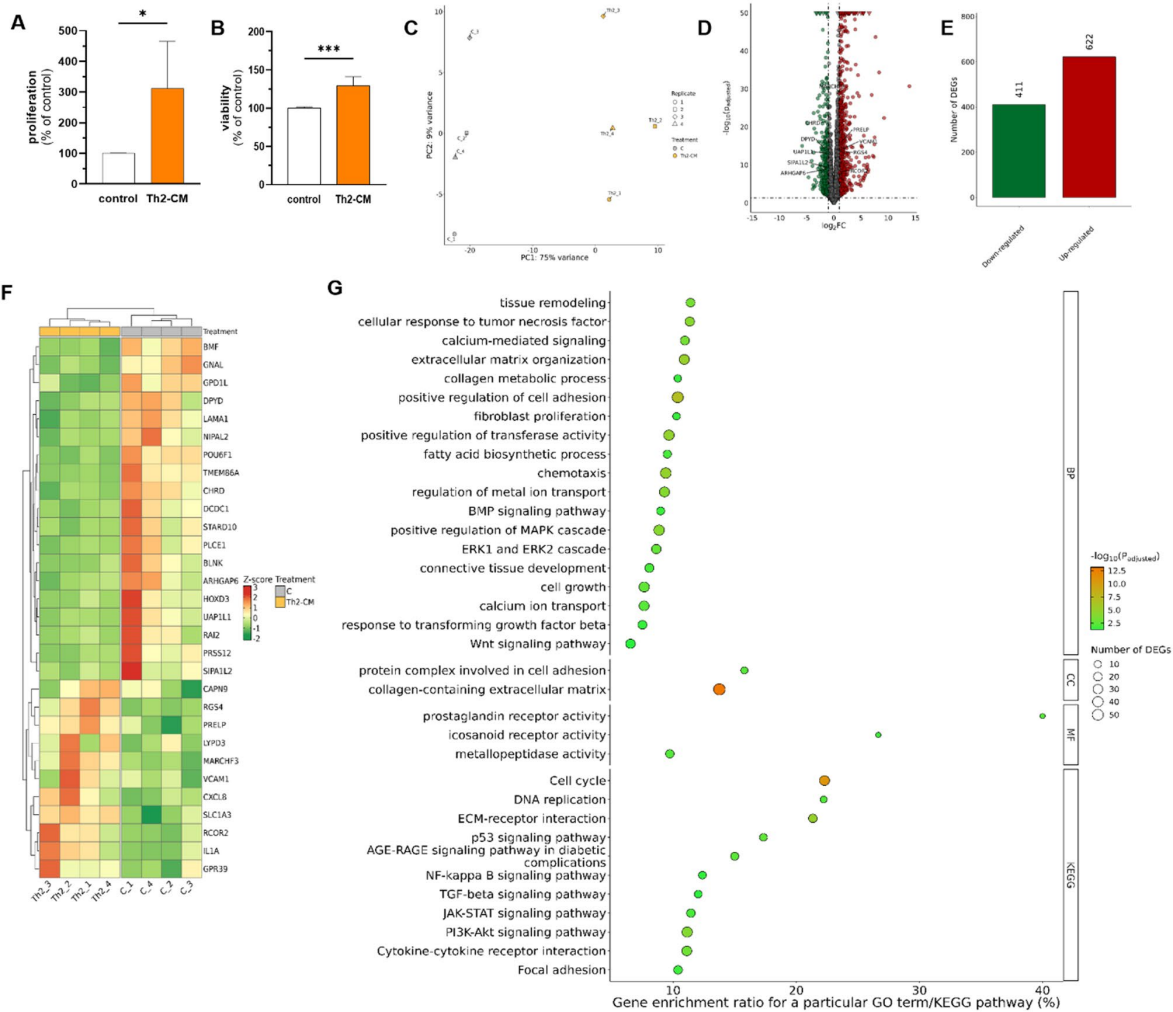
The effect of Th2-conditioned medium (Th2-CM) treatment on proliferation (**A**) and viability (**B**) of mare endometrial fibroblasts (n = 5) after 48 h. Results were calculated as the mean ± standard deviation (SD) and expressed as a percentage of the respective control group. Asterisks denote statistical differences (* p < 0.05, *** p < 0.001). The differences were determined using the unpaired Student-t test. Graphical presentation of the first (PC1) and second (PC2) principal components (PCA) of the mRNA expression pattern in mare endometrial fibroblasts (n = 4) treated with Th1-CM (**C**). The volcano plot (**D**) presents differentially expressed genes (DEGs; padjusted < 0.05 and log2FC ≥ 1.0 or log2FC ≤−1.0) which are represented by multi-coloured circles, where red colour is up-regulated genes and green down-regulated genes. (**E**) The number of identified up- and down-regulated DEGs. Heatmap (**F**) illustrates the expression profile of top 30 DEGs, selected by lowest padjusted values. The colour scale of the heatmap shows the normalized (Z-score) expression level where red blocks represent up-regulated DEGs, and green blocks represent down-regulated DEGs. C—control group (untreated cells). Selected Gene Ontology (GO) and Kyoto Encyclopedia of Genes and Genomes (KEGG) terms enriched by identified DEGs (padjusted < 0.05 and log2FC ≥ 1.0 or log2FC ≤ −1.0) in fibroblasts derived from category I endometria (n = 4) treated with Th2-CM for 48 h (**G**). The y-axis is GO/KEGG categories. The x-axis refers to GO/KEGG enrichment. BP– Biological Processes, CC—cellular components, MF—molecular functions.

The study on the effect of Th2 cell secretome (Th2-conditioned medium; Th2-CM) on the proliferation and viability of endometrial fibroblasts revealed changes similar to Th1-CM treatment. T helper 2 cell secretome increased both mare endometrial fibroblast proliferation and viability (Fig. 6A, B; p < 0.05). Moreover, Th2-CM treatment induced transcriptomic changes in mare endometrial fibroblasts, as presented in Fig. 6C-F. Furthermore, the transcriptomic study demonstrated that the Th2 cell secretome affected the expression of 1033 genes including 411 down- and 622 up-regulated in endometrial fibroblasts, as presented in the Fig. 6E. The full list of DEGs identified in this study is presented in the Supplementary Table 3. The top five upregulated DEGs were *COL6A5, ALOX15, NTRK1, CCL26*, and *CXCL9*. The most five downregulated DEGs after Th2-CM treatment were *PTGER3, GPX3, ADAMTS8, IGSF10*, and *PRLR*. Differentially expressed genes identified in fibroblasts treated with Th2-CM were then subjected to functional analyses involving the GO and KEGG database. It revealed 919 BP, 59 CC, 83 MF terms, and 21 KEGG pathways in which identified DEGs may be involved. Selected most enriched GO terms and KEGG pathways are presented in Fig. 6G. The full list of GO and KEGG terms is provided in the Supplementary Table 4.

**Fig. 7:**
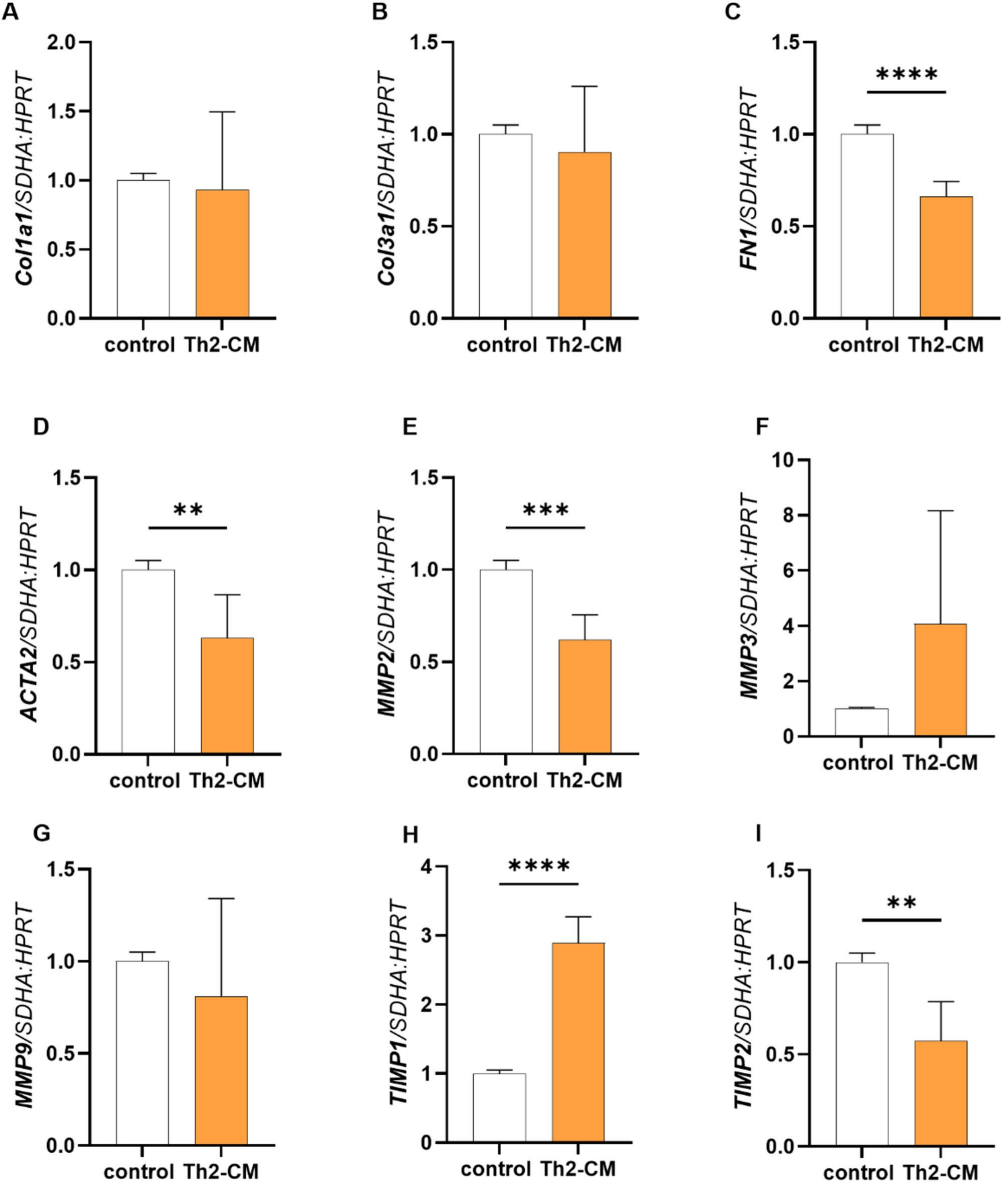
The effect of Th2 conditioned medium (Th2-CM) on mRNA transcription of *collagen (Col)1a1* (**A**)*, Col3a1* (**B**), fibronectin 1 (*FN1*; **C**)*, α-smooth muscle actin* (*ACTA2;* **D**)*, matrix metalloproteinase* (*MMP*) 2 (**E**)*, MMP3* (**F**)*, MMP*9* (G), tissue inhibitor of matrix metalloproteinase* (TIMP)1 (**H**)*,* and *TIMP2* (**I**) in fibroblasts derived category I endometria after 48 h of treatment. Results were calculated as the mean ± standard deviation (SD) and expressed as a fold change to the control group. Asterisks denote statistical differences (**p < 0.01, p < 0.001, ****p < 0.0001), as determined by unpaired Student t-test.

The effect of Th2-CM treatment on the mRNA transcription of ECM-associated markers in fibroblasts derived from category I endometria after 48 h is shown in Fig. 7. Treatment with Th2-CM decreased FN1 (Fig. 7C; p < 0.0001), *ACTA2* (Fig. 7D; p < 0.01), *MMP2 (*Fig. 7E; p < 0.001), and *TIMP2* (Fig. 7I; p < 0.01) and increased *TIMP1* (Fig. 7H; p < 0.0001) mRNA transcription in mare endometrial fibroblasts. However, no changes of *Col1a1* (Fig. 7A)*, Col3a1* (Fig. 7B)*, MMP3* (Fig. 7F)*,* and *MMP9* (Fig. 7G) mRNA transcription were observed in fibroblasts treated with Th2-CM (p > 0.05).

## Discussion

T cells appear to play an important role in the endometrium of mares, influencing the inflammatory response, which is critical in the development of endometrosis^5^. The infiltration of T cells (CD3^+^) has been observed in the endometria of mares with endometrosis^5^. T cells were highly prevalent within the fibrotic glands of the equine endometrium^5^. Hovever, to date the distribution of Th1 and Th2 cells in mare endometrium has not been determined yet. The results of this study indicate the presence of Th1 and Th2 cells at every stage of Kenney and Doig´s endometrium classification, and therefore, also in the moderate (category IIB), and in the severe stage of endometrosis (category III), with no fluctuations between endometrial categories. However, these findings may not fully capture the distribution of Th cell subsets and their cytokines in the progression of mare endometrosis, a complex condition marked by diverse characteristics, including fibrotic changes dispersed within the stroma and around endometrial glands^5,29^. Differentiating between fibrotic and unaffected endometrial regions could offer greater insights, necessitating advanced methods, such as laser microdissection, spatial proteomics, single-cell RNA sequencing, and immunohistochemistry. These approaches have proven effective in other fibrotic conditions, such as lung fibrosis in COVID-19 and liver cirrhosis^30–32^, highlighting their potential application in endometrosis research.

Interferon γ plays a pivotal role in modulating a vast array of immunological and inflammatory pathways within the human body. Significant variations in its levels have been observed between healthy and fibrotic tissues^33^. It has been demonstrated that the level of IFN-γ varies in different organs undergoing fibrosis in different species^34–36^. A positive correlation was observed between the level of IFN-γ and the development of liver fibrosis in humans^34^. In contrast, lower serum levels of IFN-γ were associated with an increased degree of pulmonary fibrosis in COVID-19 patients^35^. However, not all research results clearly indicate a correlation between IFN-γ levels and the development of fibrosis. For instance, no alteration in IFN-γ serum levels was observed in patients with systemic sclerosis^36^. In the current study, no differences were found in the concentration of IFN-γ and its receptor at both gene and protein levels between all mare endometrial categorie, including moderate and severe endometrosis stages (category IIB and III, respectively). Results of the present study are consistent with those previously published, which found no differences in the mRNA transcription of *IFN-γ* and its receptor in mare endometria during the estrous cycle^37^.

Th1 cells contribute to the development of fibrosis by promoting inflammation through the release of pro-inflammatory cytokines, including IFN-γ, TNF-α, and IL-2. These cytokines can activate macrophages and modulate fibroblast activity^15^, which may ultimately lead to the onset of tissue inflammation and heart fibrosis in mice^38^. Interferon-γ^-/-^ mice, deficient in Th1 cells have been observed to develop significantly greater lung fibrosis compared to mice expresing IFN-γ^39^. Nevertheless, Th1 cells have also been identified as a pro-fibrotic contributor in cardiac fibrosis by utilizing integrin α4 to adhere to cardiac fibroblasts and promote TGF-β production which is mediated by IFN-γ^38^. However the precise influence of Th1 cells on the development of endometrial fibrosis in the progression of mare endometrosis remains unknown, as no studies have directly addressed this issue. The results of the present study indicated that Th1-CM treatment increased both the proliferation and viability of the mare endometrial fibroblasts. The literature indicates that Th1-secreted cytokines, including IFN-γ and TNF-α, modulate the behaviour and proliferation of fibroblasts^40^. Fibroblast proliferation and metabolic activity play a crucial role in the development and progression of fibrotic disorders. In fibrotic disorders, fibroblasts are often abnormally activated and proliferate in response to various stimuli, including cytokines, growth factors, and mechanical stress^19^. This proliferation leads to an increased number of fibroblasts within the affected tissue, which then produce excessive amounts of ECM components^41^. The accumulation of ECM components consequently disrupts the normal tissue architecture and function, contributing to the stiffening and scarring that are characteristic of fibrosis.

The Th1-CM treatment changed the expression profile of genes associated with the metabolism of COLs in equine endometrial fibroblasts. Disturbances in COLmetabolism processes are key factors contributing to the development of fibrosis^42^. Results of the recent transcriptomic study on mare endometrium with endometrosis revealed alteration in the expression of genes involved in the catabolism of COLs (e.g. *MMP7*)^43^. In this study, seven other MMPs were found to be dysregulated after Th1-CM treatment: two were upregulated (*MMP23* and *MMP25*), while five were downregulated (*MMP11, MMP12, MMP13, MMP19,* and *MMMP24*). The down-regulation of these MMP expressions may indicate the disturbances of the ECM turnover and thus excessive ECM deposition. It was previously reviewed that MMPs, especially MMP12 and MMP13, may play both pro- and anti-fibrotic roles in experimentally induced fibrosis in rodents, depending on the cause of the fibrosis and the affected organ^44^. These findings implicate the importance of Th1 cells in endometrial remodeling. For example, MMP-13, also known as collagenase 3, activity allows the activation and release of active forms of TNF-α and insulin-like growth factor (IGF). The ability to activate and release TNF-α is also a characteristic of MMP-12 and MMP-7^45^. In turn, TNF-α, can modulate immune cell acitivity and function, including T cells and macrophages as well as other cells, e.g. endothelial, epithelial, smooth muscle cells, and fibroblasts, thus affecting fibrosis outcome^46^. Moreover, the importance of TNF-α in fibrotic disorders is reflected by the use of TNF-α antagonists in the treatment of inflammatory diseases with terminal fibrosis, such as rheumatoid arthritis, spondyloarthropathies, Crohn’s disease, and ulcerative colitis in humans^46^. The dysregulation of MMP expression in endometrial fibroblasts after Th1 cell secretome treatment indicates that Th1 cells may play an important role in the development of fibrosis in endometrosis progression.

Differentially expressed genes identified in fibroblasts treated with Th1-CM were also associated with such processes as ECM-receptor interaction, protein digestion and absorption, and focal adhesion. Extracellular matrix-receptor interactions are fundamental to cell signaling processes that regulate cell functions, including proliferation, migration, and differentiation. These altered ECM-receptor interactions promote the differentiation of fibroblasts into myofibroblasts^47^. Focal adhesions are specialized structures that connect cells to the ECM via integrin receptors^48^. Beyond their role as physical attachment points, they function as signaling hubs that regulate essential cellular processes such as survival, proliferation, and migration^48^. In fibrotic tissues, focal adhesions are crucial in mechanotransduction, the process by which mechanical signals from the ECM are transformed into biochemical signals within the cell. These signals sustain the activation of fibrogenic pathways, thereby advancing the progression of fibrosis^49^. Results of our recent study indicated that target genes of the microRNAs, which were found to be dysregulated in endometria with endometrosis (categories IIA, IIB, and III, compared to endometria category I) can be involved in ECM-receptor interaction^50^. These findings suggest that these processes play an important role in the development of fibrosis in the progression of endometrosis.

The findings of this study indicate that the Th1 cell secretome may modulate processes associated with the development of fibrosis in the endometrosis progression, which are not directly connected with ECM deposition. The treatment of fibroblasts with the Th1 cell secretome resulted in alterations in the expression of genes, which are involved in the metabolic processes of fatty acids. It is of great importance since excessive lipid accumulation or impaired fatty acid oxidation is linked to heightened lipotoxicity, which plays a direct role in the development of fibrosis^51^. Metabolic disruptions play a critical role in fibrotic disorders by altering cellular energy balance and promoting the activation of fibroblasts, which leads to excessive ECM deposition. These metabolic changes can exacerbate inflammation and tissue remodeling, contributing to the progression of fibrosis^52–54^. Results of our recent study revealed that DEGs identified in the endometria with endometrosis may be involved in the metabolism of carbohydrates, amino acids, proteins, and lipids^10^. Furthermore, results of the past study also demonstrated alterations in the expression of genes involved in the lipid metabolic process (e.g. *EPP6*) in endometria with endometrosis^43^. Taken together, these results indicate that changes in the cellular metabolism are present in endometria with endometrosis, but also in other tissues undergoing fibrosis. One of the top up-regulated DEGs in fibroblast after Th1-CM treatment is *15-lipoxygenase* (*ALOX15*)*.* The enzyme 15-lipoxygenase can oxidize polyunsaturated fatty acids, and is involved in the production of specialized pro-resolving lipid mediators, which, in turn, were shown to be crucial for resolving inflammation and modulating inflammatory responses^55^. However, ALOX15 is not inherently pro-resolving, since its action depends on the polyunsaturated fatty acids that are available. With DHA/EPA as substrates, ALOX15 helps generate specialized pro-resolving mediators (e.g., resolvins, protectins, maresins). With arachidonic acid, it can amplify inflammation^56,57^. Thus, substrate supply is a stronger determinant of outcome than the enzyme alone. Given that Th1-CM is rich in pro-inflammatory cytokines and can polarize fibroblasts toward a Th1-type chemokine program, a shift toward a more pro-inflammatory response is expected when Th1-CM is present^12^. The data suggest that Th1 cells, through the secretion of specific factors, may exert a significant influence on fibroblast metabolism. Furthermore, this suggests that metabolic reprogramming in the mare’s endometrial fibroblasts may be influenced by the Th1 secretome.

Notably, DEGs identified in endometrial fibroblasts after Th1-CM treatment were found to enrich the process of prostaglandin (PG) receptor activity. Prostaglandins play a role in the regulation of various physiological processes in the endometrium. These molecules are involved in managing inflammation, vascular function, and cellular responses within the endometrial tissue^58^. Thus, it seems that Th1 cells may play an important, modulative effect in mare endometrium also by affecting PG action. It has been demonstrated that during the progression of endometrosis alterations in the PG concentration, and in the expression of PG receptors occur^59^. Prostaglandins were shown to play an important role in processes related to the development of fibrosis^60^. It seems that PGs may be involved in the progression of mare endometrosis by regulating ECM-associated marker expression in endometrial fibroblasts^61^. The treatment of mare endometrial fibroblasts with PGE_2_ resulted in increased *TIMP1* mRNA transcription, as well as MMP-2 and MMP-9 concentration, whereas MMP-13 mRNA transcription and concentration were decreased after PGE_2_ treatment. In turn, the treatment of mare endometrial fibroblasts with PGF_2α_ resulted in a decrease in MMP-1 concentration and an increase in MMP-2 and MMP-13 concentration. Moreover, PGF_2α_ affected *TIMP-1* and *TIMP-2* mRNA transcription, and *COL1A1* mRNA transcription and COL1 concentration. Thus, PGE_2_ seems to be mostly anti-fibrotic, while PGF_2α_ exhibits a pro-fibrotic action in the mare endometrial fibroblasts^61^. All these results suggest that Th1 cells regulate the expression of PG receptors and Th1 may indirectly PG action, while PGs have been shown to influence the expression of ECM-related factors.

T helper 1 cell secretome affects the expression of genes, which are involved in DNA replication and cell cycle in endometrial fibroblasts. The changes in the expression of Ki-67, a marker of cell proliferation, have been demonstrated in the mare endometria with inactive endometrosis^8^. The findings indicate alterations in the proliferation and, consequently, in the cellular cycle of the endometrial cells in mares with endometrosis. The dysregulation of the gene expression associated with the cell cycle has been demonstrated to contribute to the development of renal fibrosis^62^. Moreover, changes in the expression of genes related to the cell cycle were shown in the lungs of idiopathic pulmonary fibrosis (IPF) patients^63^. Furthermore, the alterations in DEGs associated with cell viability and cell cycle were previously identified in mare category IIB endometrium, with moderate endometrosis, compared to healthy category I endometrium^10^. The data suggest that Th1 cells, via their secretome, may be involved in the regulation of processes that are important in the development of endometrosis. However, further studies are necessary to elucidate the effect of Th1 cells on the cellular cycle in the mare endometrium.

Fibrosis develops when the balance of ECM turnover is disturbed, leading to excessive ECM deposition without proper resolution. This imbalance leads to the disruption of tissue architecture, thereby contributing to the progression of fibrotic diseases. The next step was to investigate the effect of Th1 cell secretome on the mRNA transcription of selected ECM-associated markers in endometrial fibroblasts, focusing on ECM components like COLs and fibronectin (FN), the myofibroblast marker ACTA2, and enzymes involved in ECM turnover, such as MMPs and their tissue inhibitors. The treatment of fibroblasts with Th1 cell secretome decreased *FN1, ACTA2, MMP2* and *TIMP2* mRNA transcription, and increased *TIMP1* mRNA transcription. The decrease in the mRNA transcription of ECM-associated factors (*FN1, ACTA2*) may suggest the anti-fibrotic action of the Th1 cell secretome. The expression of *ACTA2* increased in the mare endometrium category IIB, compared to category I endometrium^64^. Thus, Th1 cells may exert an anti-fibrotic action by inhibiting myofibroblast differentiation. Moreover, the decrease in *FN1* expression also suggests that Th1 cells may attenuate processes contributing to fibrosis, as targeting of FN1 expression has been proposed as a treatment option for cardiac fibrosis^65^. On the other hand, the increase in *TIMP1* mRNA transcription may indicate the pro-fibrotic effect of Th1 cells. Increased levels of TIMP 1 may result in the inhibition of MMP-9 activity, which main role is to degrade ECM, and this, in turn, leads to excessive ECM deposition. However, a study on liver fibrosis showed that the presence of TIMP1 may contribute to the progression of fibrosis, but is not necessarily^66^. Moreover, our results show that Th1 cell secretome decreased *MMP-2* mRNA transcription in endometrial fibroblasts. Although MMP-2 activity was previously shown to be crucial in the TGF-β2-induced matrix contraction in human lens epithelial cell-line^67^, the reduced MMP-2 activity was shown to promote cardiac fibrosis, as shown in the experimental model of diabetic cardiomyopathy^68^. In addition, Th1-CM reduced *TIMP-2* mRNA transcription, whose pivotal role is to inhibit MMP-2 activity^69^ thus suggesting that Th1 may play an anti-fibrotic role in mare endometrial fibroblasts. However, further studies on the effect of Th1 on the protein abundance of selected ECM-associated markers are essential to determine if Th1 cells play pro- or anti-fibrotic roles on mare endometrial fibroblasts.

In some fibrotic diseases, T helper 1 cells may predominantly depict anti-fibrotic properties^27^. Nevertheless, in the present study, the findings indicate that Th1 cells may be involved in the regulation of processes associated with the development of fibrosis in the progression of equine endometrosis. These cells exert a direct effect on equine endometrial fibroblasts, increasing their proliferation. Furthermore, they modulate the expression of genes involved in a multitude of biological processes and signaling pathways including cell cycle, metabolism, and those crucial for ECM deposition, such as ECM-receptor interaction, protein digestion and absorption, focal adhesion, and collagen binding. However, further studies are required to ascertain whether the action of Th1 cells is pro- or anti-fibrotic.

Th2 cells are primarily considered pro-fibrotic due to production of cytokines like IL-4, IL-5, and IL-13, which promote the activation of fibroblasts and enhance COL production^22,70–73^. These cytokines drive the deposition of ECM, contributing to tissue scarring and fibrosis. T helper 2 cell-mediated responses are associated with chronic inflammation and impaired tissue repair, which are key processes in the development and progression of fibrotic diseases. However, there are no studies concerning the effect of Th2 cells on fibrosis progression in mare endometrosis. The mechanisms by which Th2 cells may contribute to the development of fibrosis involve the deposition of ECM by fibroblasts in response to various cytokines and growth factors produced by fibroblasts^74^. Despite the absence of studies examining the effect of Th2-CM on fibroblast proliferation and metabolic activity, there is a body of evidence on Th2-specific cytokines that corroborates the findings of this study. The treatment with IL-4 and IL-13 (10 ng/mL) increased the metabolic activity of normal human lung fibroblasts^75^. Furthermore, IL-4 and IL-13 have been demonstrated to stimulate the proliferation and production of ECM components by hepatic fibroblasts in mice^72^. Similarly, another Th2-secreted cytokine—IL-5 has been shown to increase the proliferation of asthmatic fibroblasts^76^.

In the development of fibrotic disorders, IL-4 levels are often elevated which, in turn, favours fibrosis development^77^. An increase in IL-4 gene expression was specifically observed in CD4 + T cells isolated from irradiated lungs, suggesting a Th2-dominated T cell response^16^. Furthermore, elevated tissue levels of IL-4 were observed in a rodent model of experimentally induced lung fibrosis^78,79^ as well as in the IPF patients^49,80^. In systemic sclerosis patients, IL-4 blood levels were found to be higher when compared to healthy individuals^36^. Additionally, elevated levels of IL-4 in blood serum were observed in liver fibrosis, particularly in advanced fibrosis accompanying chronic hepatitis C^81^. In fibrotic disorders, IL-13 levels are typically elevated and contribute significantly to the pathological processes that lead to tissue and fibrosis. More precisely, the level of IL-13 in blood plasma was significantly elevated in patients diagnosed with IPF^82^ and asthma-related lung fibrosis^83^. Moreover, the level of IL-13 in blood plasma was significantly higher in patients with systemic sclerosis and localized scleroderma compared to healthy controls. These elevated levels were positively correlated with the severity of skin fibrosis and the presence of inflammatory biomarkers^84–86^. Elevated IL-13 serum levels were also associated with increased cirrhosis-associated liver fibrosis in patients^86^ and in non-alcoholic steatohepatitis in rat models^87^.

The results of the transcriptomic study on the effect of Th2-CM treatment on mare endometrial fibroblasts indicate that Th2 cells can affect multiple processes associated with the development of fibrosis. T helper 2 cell secretome affected the expression of genes involved in tissue remodeling and inflammatory response to wounding. Furthermore, identified DEGs may participate in collagen-containing extracellular matrix and adherens junction. Proper tissue remodeling is essential for maintaining tissue structure and function during the healing processes. The inflammatory response is a critical element of tissue remodeling, initiating the repair process by removing damaged cells and promoting the recruitment of immune cells^88^. However, if the inflammatory response is prolonged or dysregulated, it can disrupt normal remodeling and thus contribute to excessive ECM deposition. T helper 2 cells may not only induce and modulate a type 2 immune response, which is crucial for fibrosis outcome^89^, but also affect the expression of genes directly constituting ECM, including COLs and proteins involved in cell-to-cell adhesion, e.g. cadherins.

T helper 2 cell secretome treatment resulted in altered expression of genes involved in e.g. NF-κB signaling pathway. The nuclear factor NF-κB pathway has traditionally been recognized as a key proinflammatory signaling pathway, predominantly due to its function in regulating the expression of proinflammatory genes including cytokines, chemokines, and adhesion molecules^90^. The NF-κB signaling pathway has previously been identified as an important signaling pathway in numerous fibrotic disorders^91–93^. A recent study reported changes in the expression of selected genes of NK-κB1 signaling pathway in mare endometria with endometrosis^94^. The findings of this study along with available literature data suggest that Th2 cells may modulate processes related to the development of endometrosis by regulating the expression of components of NF-κB signaling pathway. Differentially expressed genes identified in endometrial fibroblasts after Th2-CM treatment were annotated to the TGF-β signaling pathway. Transforming growth factor-β1 is widely recognized as the most potent profibrotic cytokine, playing a central role in the development and progression of fibrosis across various tissues. It has been demonstrated that there is a positive correlation between the levels of TGF-β1 in mare endometria and the progression of endometrosis^95^. Furthermore, TGF-β1 was observed to enhance the mRNA transcription of *COL1A1*, *COL3A1*, *FN1*, and *ACTA2*, as well as their protein abundance, in a manner that was both time and dose dependent in endometrial fibroblast^64^. Additionally, TGF-β1 was demonstrated to stimulate the proliferation of mare endometrial fibroblasts *in vitro*^64^.

The study on the effect of Th2 cell secretome on the expression of ECM-associated genes in endometrial fibroblasts revealed changes similar to Th1-CM treatment on the fibroblasts. T helper cell secretome decreased *FN1, ACTA2, MMP2* and *TIMP2* mRNA transcription, and increased *TIMP1* mRNA transcription in endometrial fibroblasts. The reduction of *FN1* and *ACTA2* suggests that Th2-CM may also exhibit anti-fibrotic action in mare endometrial fibroblasts. The reduction in the expression of these ECM components could be associated with an impaired fibrotic response to Th2-CM treatment. Fibronectin was suggested as a target in the treatment strategy for liver and heart fibrosis^65,96^, indicating its importance in the development of fibrotic diseases. However, a study on hepatic stellate cells, which are key cells in liver fibrosis, indicated that FN may regulate the expression of growth factors, including TGF-β. The study on FN-depleted mice shows that lack of FN increases TGF-β levels and signaling, enhances hepatic stellate cell proliferation and activation^97^. These results indicate that FN may also exhibit anti-fibrotic action by ameliorating processes associated with the excessive ECM components deposition. However, the action of FN may be both species and tissue-dependent. Thus, further studies are needed to establish the role of FN in mare endometrial fibroblasts. A reduction in *MMP2* mRNA transcription implies a decrease in ECM degradation activity. However, in the context of fibrosis, this could be complex because while reduced MMP activity might slow ECM remodeling, it could also lead to a decrease in the resolution of fibrosis if not properly balanced^44^. The shift in *TIMP* mRNA transcription suggests a change in the regulation of ECM turnover. Increased *TIMP1* mRNA transcription alongside decreased *TIMP2* mRNA transcription might indicate a more complex regulation of MMP expression, possibly leading to altered ECM dynamics that could affect the progression or resolution of fibrosis^44^. All these results indicate that Th2 cells can directly affect fibroblast properties and expression of ECM-associated markers. Moreover, the Th2 cell secretome may regulate the expression of genes involved in multiple signaling pathways and cellular processes, which were described as important in the development of mare endometrosis, but also in the development of other fibrotic disorders. However, further studies are required to clarify whether Th cells contribute to a pro-fibrotic response in the mare endometrium.

We observed that fibroblasts exhibited similar responses when treated with Th1-CM and Th2-CM. In vitro polarization of Th0 cells into Th1 or Th2 includes TCR stimulation and defined cytokine cocktails (e.g., IL-2, IL-12, IFN-γ vs IL-4) with opposing cytokine blockade, yielding highly polarized, relatively homogeneous populations. However, in contrast, in vivo differentiation is shaped by antigen dose and persistence, APC subtype/co-stimulation, tissue cues, and cross-talk with other lymphocytes, producing heterogeneous and plastic Th states including hybrid T-bet⁺GATA3⁺ cells that shift with context^98–100^. As a result, in vitro. generated Th1 and Th2 can share overlapping mediator profiles or retain intermediate phenotypes; carryover of exogenous cytokines may also dominate readouts. As a result, fibroblasts exposed to these in vitro differentiated Th1 or Th2 cells may respond in similar ways (e.g., changes in transcriptome), masking differences that might be clearer in vivo. These limitations argue for rigorous T cell phenotyping and confirmation using physiological parallel cultures or ex vivo T cells as a direction for further research.

The current study found that the secretome of both Th1 and Th2 cells increased fibroblast proliferation and induced significant changes in the mRNA transcriptome of mare fibroblasts derived from category I endometrium, cultured in vitro. These changes involved ECM-associated markers and pathways related to tissue remodeling, ECM organization, and signaling pathways like TGF-β and NF-κB suggest that the role of these cells in the processes associated to the development of endometrial fibrosis in the progression of endometrosis. These findings mark a pivotal step toward unraveling the intricate interplay between Th1 and Th2 cells and fibroblasts shedding light on their potential role in driving fibrosis progression in the development of endometrosis.

## Materials and methods

### Experimental design

**Fig. 8:**
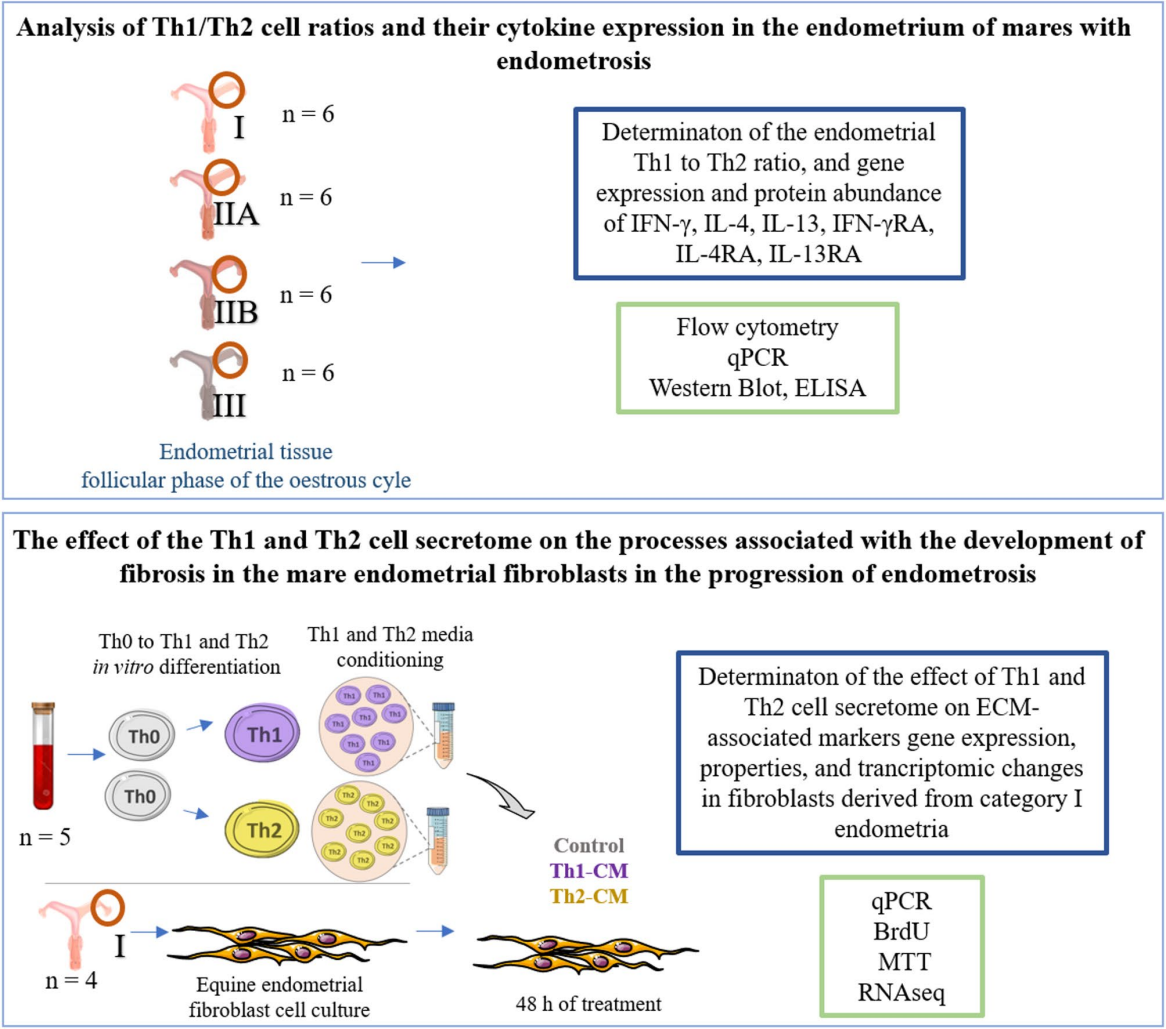
Experimental design.

The experimental desing is presented in Fig. 8. The study investigates the Th1/Th2 cell ratios and IFN-γ, IL-4, IL-13, and their respective receptor expression in the endometrium of mares with endometrosis. Endometrial tissue samples were collected from mares in categories I, IIA, IIB, and III endometria of endometrosis (n = 6/category, ∑n = 24). The objective was to determine the Th1/Th2 cell ratio and analyze the mRNA transcription and concentration of cytokines (IFN-γ, IL-4, IL-13) and their receptors (IFN-γR, IL-4R, IL-13R). Methods employed include flow cytometry, qPCR, Western blot, and ELISA. Additionally, an in vitro model was used to examine the effect of Th1 and Th2 cell secretomes on endometrial fibroblasts isolated from category I endometria (n = 4). Th0 cells were differentiated into Th1 and Th2 cells (n = 5), and Th1 and Th2 cell secretomes (Th1-CM and Th2-CM) were collected and used to treat equine endometrial fibroblast. The objective was to evaluate the impact of Th1-CM and Th2-CM on the ECM-associated marker mRNA transcription, fibroblast properties, and transcriptomic changes, using methods such as qPCR, BrdU assay, MTT assay, and RNA sequencing.

### Tissue collection for fibroblast isolation and histological analysis

Procedures were reviewed and accepted by the Local Ethics Committee for Experiments on Animals in Olsztyn, Poland (Approval No. 27/2022). Thirty-three clinically healthy, normally cycling mares (weighing 500 ± 100 kg) between age 2–20 were used in this study. The mares were healthy as stated by the official governmental veterinary inspection. The animals were slaughtered to obtain meat as part of routine breeding as slaughter animals. Endometrial tissue samples were collected from mare endometrium. The endometria were collected 5 min after slaughter. The follicular phase of the oestrous cycle was identified, based on the macroscopic observation of ovaries, characterized by the absence of an active corpora lutea and the presence of ovarian follicles > 35 mm in diameter^101^. Endometrial tissue, from the uterine tubal horn, located on the side of the active ovary was excised, rinsed with cold sterile RNase-free saline solution, and placed in liquid nitrogen for RNA and protein extraction. Furthermore, two tissue sections (~ 1.5 cm^2^) from each uterus were immediately placed in 4% paraformaldehyde and further processed for for haematoxylin–eosin staining and histological examination. Additionally, the uterine horns were transported on ice in sterile saline or Roswell Park Memorial Institute (RPMI) 1640 Medium (RPMI 1640 medium; R6504, Sigma) to the laboratory, for further isolation of fibroblasts and T cells, respectively. After haematoxylin–eosin staining, endometria were retrospectively assigned to Kenney and Doig classification^2^. This classification considers primarily the presence of inflammation and fibrosis, but also endometrial atrophy and dilatation of lymphatic vessels. Endometrium without pathological changes was classified as category I. Category IIA includes endometrium with mild to moderate inflammation, with few dilated lymphatic vessels. A slight level of fibrosis impacts specific branches of the endometrial glands, and there are no fibrotic gland observed in four neighbouring visual fields. Category IIB encompasses a moderately inflamed endometrium featuring numerous dilated lymphatic vessels. Fibrosis is more pronounced, in comparisson to category IIA, presenting 2–4 fibrotic glandular nests in four neighbouring visual fields. These alterations involve up to 60% of the uterine glands. Category III describes an endometrium characterized by significantly dilated lymphatic vessels. The fibrosis is extensive, with five or more fibrotic glands identified in four neighbouring visual fields. These changes affect more than 60% of the uterine glands, marking the most severe stage of endometrosis^102^.

### The isolation and culture of fibroblasts from mare endometrium

The fibroblasts were isolated from category I mare endometria (n = 4). Prior to the fibroblast isolation, the uterine horns were washed with sterile phosphate-buffered saline (PBS, P4417; Sigma), and the epithelial cell layer was scrapped off with a scalpel blade. Further, the endometrial layer was dissected from the underlying myometrium using scissors and submerged in a digestion solution: Hank’s balances salt solution (HBSS; H1387, Sigma) containing 0.1% bovine serum albumin (BSA; A2153, Sigma), collagenase I (0.05%; C2674, Sigma), DNase I (0.05%; 11,284,932,001, Roche), and cut into small pieces. The digestion was performed in 37 ℃ with constant stirring. After 45 min, cells were filtered through a series of 100, 70, and 40 µm cell strainers, and centrifuged at 100* × g* for 10 min at 4℃. Then, the supernatant was discarded and the cell pellet was suspended in 2 mL of red blood lysis buffer (R7757, Sigma) and mixed for 1 min, and then washed with HBSS containing 0.1% BSA. After centrifugation, cells were suspended in Dulbecco’s Modified Eagle’s Medium/Nutrient Mixture F-12 Ham (DMEM/Ham’s F-12 medium; D8900, Sigma) containing 10% fetal bovine serum (FBS; F9665, Sigma) and counted using a haemocytometer. The viability of endometrial cells was higher than 95% as assessed by the trypan blue exclusion test (Trypan Blue Solution (0.4%, 1,520,061, Gibco). The cells were counted using a Countess 3FL (Thermofisher) and seeded at a density of 5 × 10^5^viable cells/mL and cultured at 38.0 °C in a humidified atmosphere of 5% CO_2_ in the air. To purify the fibroblast population, 18 h after plating, by which time selective attachment of fibroblasts had occurred, the medium was changed to fresh fibroblast culture medium—BMTM Basal Medium (CC-3131, Lonza) and FGMTM-2 SingleQuotsTM supplements (CC-4126, Lonza) including 2% FBS, insulin, human fibroblastic growth factor (hFGF)-B, gentamycin-amphotericin (GA), and 100 ng/mL ascorbic acid (A4403, Sigma).

The fibroblast homogeneity was confirmed using immunofluorescent (IF) staining for vimentin. The fibroblast population exhibited a purity of approximately 96% following the isolation process. Once the cells had reached 90% of confluency, they were cryopreserved. For this purpose, the cells were washed with sterile PBS and detached from cell culture dish using 0.025% trypsin–EDTA solution (T4049, Sigma). To inhibit trypsin activity, medium containing 10% FBS was added after detachment. Then, cells were centrifuged at 200 × *g* for 10 min and counted using a haemocytometer. Subsequently, 1 × 10^6^ cells were suspended in 1 mL of cell freezing medium, comprising 84% of inactivated FBS and 16% DMSO (D2650, Sigma) and frozen. The samples were stored at − 80 ℃ until further experiments.

### Mare endometrial fibroblast cell culture and treatment

Thawed fibroblasts (n = 4 endometria) were seeded on T75 cm^2^ flasks and cultured at 38.0 °C in 5% CO_2_. After reaching 90% of confluence, fibroblasts were passaged and seeded on 6-well plates for RNA analysis and on 96-well plates for cell proliferation and viability assays. The experiments were performed in three technical replicates. After reaching 80% confluence, the culture medium was replaced with medium alone, Th1-CM or Th2-CM. After 48 h of treatment, the cells were washed with sterile PBS (P4417; Sigma-Aldrich, St. Louis, MO, USA), and lysed with TRI Reagent (T9424; Sigma-Aldrich, St. Louis, MO, USA) for RNA and stored in -80°C for subsequent RNA extraction and subsequent analyses.

### Cell proliferation assay—BrdU assay

The proliferation of mare endometrial fibroblasts was assessed using an ELISA bromodeoxyuridine (BrdU) incorporation assay (11,647,229,001, Roche), which is based on BrdU incorporation, as a thymidine analogue, into nuclear DNA of proliferating cells. To perform the BrdU assay, mare endometrial fibroblasts were seeded into 96-well plates, at a concentration of 1 × 10^4^ cells per 100 µL of medium per well. After reaching the desired confluence, cells were treated for indicated periods of time, and then labelled with BrdU (final concentration 10 µM) for two hours. The labelling medium was then removed by tapping off. To fix the cells, 200 µl of FixDenat solution was added and incubated for 30 min at 25 ℃. Subsequently, the FixDenat solution was removed thoroughly by flicking and tapping. Thereafter, 100 µl of antibody-conjugate (anti-BrdU-POD working solution) were added per each well, followed by incubation for 90 min at 25 ℃. The antibody-conjugate was then removed by flicking off and rinsed three times with 200 µl of washing solution (PBS 1X).per well. After the third washing, the plates were tapped to remove any residues of the washing solution and 100 µl of substrate solution was added per well, followed by 30 min of incubation at 25 ℃. After the incubation period, the colour development was observed, and the absorbance of the samples was measured in an ELISA reader (BioTek Microplate Reader, Epoch) at the wavelength 370 nm. The absorbance value of the control group (untreated cells) was established as 100%, while that of the treated cells was calculated as a ratio to the control cells × 100%.

### Cell viability and metabolic activity assay—MTT assay

The viability (metabolic activity) of fibroblasts (n = 4) was determined using the MTT assay (Mosmann, 1983). The MTT kit (TOX1, Sigma), which was employed to ascertain the viability and metabolic activity of cells. This assay is based on the reduction of MTT (3-[4,5- dimethylthiazol-2-yl]-2,5-diphenyl tetrazolium bromide), a yellow tetrazolium salt, by metabolically active cells into purple, water-insoluble formazan crystals^103^. Viable cells contain NADH-dependent oxidoreductase enzymes, which reduce the MTT to water-insoluble formazan, which is further solubilized in acidic isopropanol (solvent), and its concentration can then be detected spectrophotometrically, at a wavelength of 570 nm.

To perform the MTT assay, mare endometrial fibroblasts were seeded at a concentration 1 × 10^4^ cells per 100 µl of medium per well into 96-well plates. Once the cells had reached the desired confluence, cells were treated. After treatment, the MTT solution was added to the wells reaching a concentration of 0.5 mg of MTT per 1 mL of medium. The cells were further incubated for three hours under normal growth conditions (38 ℃, 5% CO_2_). Following the incubation, which resulted in the transformation of yellow MTT into purple formazan crystals, 100 µl of MTT solubilization solution was added to each well, followed by thorough mixing by pipetting. The concentration of formazan was then measured using a BioTek Microplate Reader (Epoch) at a wavelength of 570 nm. The absorbance value of the control group (untreated cells) was established as 100%, while that of the treated cells was calculated as a ratio to the control cells × 100%.

### Isolation of T cells from mare’s endometrium

The T cells were isolated from endometrium of mares in the follicular phase of the oestrous cycle (n = 6 per category, ∑n = 24). Prior to T cell isolation, the uterine horns were washed with sterile PBS and the endometrial layer was dissected from underlying myometrium using scissors and submerged in digestion solution, which consisted of HBSS containing 0.1% BSA collagenase IV (0.05%, C5138, Sigma), DNase I (0.05%), and then cut into small pieces. The digestion was performed in 37 ℃ with constant stirring. After 45 min, cells were filtered using successively 100, 70, and 40 µm cell strainers, and centrifuged at 100 × *g* for 10 min at 4 ℃. Then, the supernatant was discarded and the cell pellet was suspended in 20 mL of HBSS containing 0.1% BSA. Then, 20 mL of cell suspension was gently placed onto 20 mL of Ficoll-Paque Plus (GE17-1440, Cytiva) in 50 mL centrifuge tubes, followed by centrifugation at 400 × *g* for 40 min at 18 ºC, with the acceleration and deceleration value at the minimum. Subsequently, the upper layer of cells was transferred to a new, sterile tube, and washed twice with HBSS containing 0.1% BSA at 500 × g for 10 min at 18 ºC. After centrifugation, cells were counted using a haemocytometer and suspended in a cell freezing medium, containing 90% of FBS and 10% of DMSO^104^. The concentration of cells in the cell freezing medium was 1 × 10^7^ cells/mL^104^. Isolated cells were stored at -80 ℃ until subsequent analysis was conducted using a flow cytometer.

### Mare’s peripheral blood collection

The study was conducted in December 2022 and January 2023. Mares

(n = 5; weighing 500 ± 100 kg) at age 2–8 years were used in this study. All mares were clinically healthy, as confirmed by veterinary inspection and individual mare health records. The experimental protocol was approved by the Local Ethics Committee for Experiments on Animals in Olsztyn, Poland (Approval No. 27/2022). All methods were carried out in accordance with relevant guidelines and regulations. All methods are reported in accordance with ARRIVE guidelines for the reporting of animal experiment. To exclude the possible influence of fluctuations in ovarian steroid characteristics oestrous cycle, T cells were isolated from non-cycling mares during the winter months. Mare’s genital tract was examined by ultrasonography to confirm anoestrous by veterinarian. Moreover, a clinical exam including physical examination, heart rate, and rectal temperature was performed to ensure that the horses were healthy. Jugular peripheral blood (15 mL) was collected from the mare (n = 5) by a veterinarian. Then, the blood was transferred to a 50 mL tube, mixed with an anticoagulant, containing ethylenediaminetetraacetic acid (EDTA, 0.38 M; 879,810,112, POCH) and acetylsalicylic acid (55 mM; 2,091,299, Polpharma) and transported to the laboratory.

### Isolation of mare’s peripheral blood mononuclear cells

For T cells differentiation, T cells were isolated from mare peripheral blood mononuclear cells (PBMCs; n = 5). For T cells isolation, 15 mL of blood with anticoagulant and 15 mL of sterile-filtered HBSS containing BSA (0.1%) were mixed using a pipette. Then, HBSS-diluted blood was gently placed onto 20 mL of Ficoll-Paque Plus in 50 mL centrifuge tubes, followed by centrifugation at 400 × *g* for 40 min at 18 ºC, with the acceleration and deceleration value at the minimum. Subsequently, the upper layer of cells was transferred to a new, sterile tube, and washed twice with HBSS containing 0.1% BSA at 500 × g for 10 min at 18 ºC. Then, cells were counted using a haemocytometer and suspended in a cell freezing medium, containing 90% of FBS and 10% of DMSO. The concentration of cells in the cell freezing medium was 1 × 10^7^ cells/mL^104^. Isolated peripheral blood mononuclear cells (PBMCs) were stored at − 80 ℃ until subsequent Th1 and Th2 differentiation.

### Th1 and Th2 cell differentiation

T helper naïve cells (Th0) were separated from PBMCs, using a negative selection kit EasySep™ Human Naïve CD4 + T Cell Isolation Kit (19,555, STEMCELL technologies). For this purpose, PBMCs (n = 5; 2 × 107 cells) were thawed by placing them at 37 ℃ for 2 min. Subsequently, the cells were transferred into 50 mL tubes, containing T cell culture medium: RPMI 1640 medium containing 10% FBS, 1% antibiotic–antimycotic solution (AA; A5955; Sigma), 1% of Non-Essential Amino Acids Solution (NEAA; 11,140,050, Gibco), 50 nM of 2-mercaptoethanol (31,350,010, Gibco), and 10 ng/mL ascorbic acid. Then, cells were centrifuged at 500 ×*g* for 10 min at 18℃. After centrifugation, the supernatant was removed, and the cell pellet was suspended in RPMI 1640 medium containing 10% FBS, 1% antibiotic–antimycotic solution, 50 nM of 2-mercaptoethanol and 100 ng/mL ascorbic acid. Then, cell quantity and viability were assessed using Trypan blue exclusion test. The viability of thawed cells was over 95%.

Undifferentiated Th (Th0) cells were isolated using EasySep™ Human Naïve CD4 + T Cell Isolation Kit (19,555, STEMCELL technologies), according to manufacturer’s instructions. For the selection of Th0 cells, 18 × 10^6^ of thawed PBMCs were placed in a new 15 mL tube and centrifuged at 500 × *g* for 10 min at 18 ℃. Then, the cell pellet was suspended in 800 µl of Easy Sep buffer (20,144, STEMCELL technologies). Next, 40 µl of EasySep™ Biotinylated AntiCD45RO Antibody and 40 µl of EasySep™ Human Naïve CD4 + T Cell Isolation Cocktail were added and mixed by pipetting. After 5 min of incubation, 40 µl of EasySep™ Dextran RapidSpheres™ were added, and the mixture was incubated for additional 5 min at room temperature. Following incubation, Easy Sep buffer was added to top up the sample to 2.5 mL and mixed by gently pipetting up and down 3 times. Then, the 15 mL tube was placed twice onto the magnet and incubated for 5 min, followed by collection of supernatants containing Th0 cells. Subsequently cells were washed, counted, and 6 × 10^6^ Th0 cells derived from each mare were transferred into new 15 mL tubes, suspended in 12 mL of T cell culture medium to obtain cell concentration at 0.5 × 10^6^ cells/mL.

Then, Th0 cells were differentiated in vitro to Th1 or Th2 cells. For Th1 and Th2 differentiation, to each tube, containing 5 × 10^5^ Th0 cells/mL, appropriate treatment was added, as depicted in Table 1^105,106^. The medium for Th1 cells differentiation contained anti-CD28, anti-IL-4, IL-12 and IL-2, whilst Th2 cells differentiating medium contained anti-CD28, anti-IFN-γ, IL-4 and IL-2 (Table 1). Then, cells were transferred to a 48-well plate, previously coated with anti-CD3ε antibodies (145-2C11, Invitrogen). The coating of plates was performed the day before differentiation protocol. Coating of a culture dish was done by adding 500 μL of anti-CD3ε antibodies at the concentration of 1 μg/mL^105^ in Dulbecco’s Phosphate Buffered Saline (DPBS; D5652, Sigma) per a well of 48-well cell culture dish. The plates were then sealed with parafilm and incubated at 4 ℃ overnight, and then a coating solution was aspirated.

**Table 1:** The concentration of stimulators used for Th1 and Th2 cells differentiation.

	anti-CD28	anti-IL-4	anti-IFN-γ	IL-12	IL-2	IL-4
	eBioscience; cat. no 16–0281	eBioscience cat. no 14–7041	eBioscience cat. no 14–7311	Bon-Opus cat. no CM39	Kingfisher Biotech cat.no RP0006E	Kingfisher Biotech cat. no RP0003E
	concentration (ng/ml)					
Th1	1000	1000	–	10	10	–
Th2	1000	–	5000	–	10	20

In order to differentiate Th0 to Th1 and Th2 cells, Th0 cells were cultured in RPMI 1640 medium containing 10% FBS, 1% AA, 1% NEAA, 50 nM of 2-mercaptoethanol, and 100 ng/mL ascorbic acid in the presence of stimulators, Th1: anti-CD28 eBioscience, 16–0281; 1000 ng/mL; anti-IL-4 eBioscience, 14–7041, 1000 ng/mL; IL-12 Bon-Opus, CM39, 10 ng/mL; IL-2 Kingfisher Biotech RP0006E, 10 ng/mL; TH2: anti-CD28 eBioscience, 16–0281; 1000 ng/mL; anti-IFN-γ eBioscience, 14–7311, 5000 ng/mL, IL-2 Kingfisher Biotech RP0006E, 10 ng/mL; IL-4 Kingfisher Biotech, RP0003E, 20 ng/mL). T helper cells were cultured for a period of six days at 38 ℃, 5% CO2 and humidity. Every two days, half the volume of the medium was replaced with medium containing the indicated concentrations of treatments. The efficiency of Th1 and Th2 cell differentiation assessed using flow cytometry (FC) was around 78% and 85% for Th1 and Th2 cells, respectively.

### Obtaining of Th1 and Th2 conditioned media

Following a six days of T cell differentiation, Th1 and Th2 cells were collected, washed with sterile-filtered PBS and suspended in RPMI 1640 medium containing 5% FBS, NEAA, 2-mercaptoethanol and ascorbic acid, at a concentration 0.5 × 10^6^ cells/mL for the purpose of obtaining Th1- and Th2-CM, respectively. Then, Th1 and Th2 cells were transferred into fresh 48-well plates, previously coated with anti-CD3ε (5 µg/mL in DPBS) and anti-CD28 (5 µg/mL in DPBS) antibodies^107^. Subsequently, the Th1 and Th2 cells were incubated for 24 h to allow conditioning of the media. Th1-CM and Th2-CM were then collected and centrifuged at 1000 ×*g* for 10 min. In order to obtain a medium from different biological replicates (n = 5), obtained media were pulled in equal ratios between biological replicates, and stored at -80℃ until fibroblast treatment. Moreover, to obtain appropriate control of Th-CM, an equal amount of RPMI 1640 medium containing 5% FBS, NEAA, 2-mercaptoethanol and ascorbic acid (100 ng/mL) was placed at − 80 ℃ for further experiments.

### Flow cytometry

For FC analysis, cells (Th cells isolated from endometrium and differentiated Th1 and Th2 cells) were washed in PBS and suspended in RPMI 1640 medium containing 10% FBS, containing Phorbol 12-myristate 13-acetate (PMA, P1585, SIGMA) at a concentration of 50 ng/mLmL and calcium ionophore—ionomycin (IAA, I9657, Sigma) at a concentration of 1 µg/mL for 2 h and monensin solution (00-4505-51, eBioscience) at a final concentration 2 uM, for additional 3 h. Both PMA and IAA are commonly used for inducing cytokine production in assays requiring the intracellular detection of cytokines in immune cells. Monensin is an inhibitor of intracellular protein transport. The incubation of cells with monensin results in the accumulation of proteins within the endoplasmic reticulum. The addition of monensin to differentiated T cells results in enhanced detection of intracellular cytokines by FC. Subsequently, the presence of Th cells markers: CD3 and CD4, as well as cytokines secreted by Th1 (IFN-γ) and Th2 (IL-4), was confirmed by FC based on the protocols described recently^106,108^. Staining of CD3 + , CD4 + and IFN-γ or IL-4 positive cells was performed using Leucoperm (BUF09B, Bio-Rad) according to the manufacturer’s instructions. Briefly, 1 × 10^6^ cells were suspended in 90 µl of PBS containing 1% BSA and 10 µl of CD4 antibodies conjugated with PE (MA5-28,356, Thermo Fisher Scientific) was added. The suspension was incubated at room temperature for 1 h. After incubation, cells were washed with PBS containing 1% BSA. Then, supernatant was removed and cells were suspended in 100 µl of fixation buffer (Reagent A), and incubated for 15 min at room temperature, followed by washing with PBS containing 1% BSA. Subsequently, the supernatant was removed and cells were suspended in permeabilization medium (Reagent B), 10 µl od anti-CD3 antibodies conjugated with PB fluorochrome (MCA1477, BioRad), 10 µl of anti-IFN-γ antibodies conjugated with Alexa Fluor 647 fluorochrome (MCA1783A647, Bio-Rad), 20 µl of anti-IL-4 antibodies conjugated with Alexa Fluor 488 fluorochrome (53-7049-42, eBioscience) were added. Then, cells were incubated for 1 h, at room temperature in darkness. After incubation, cells were washed with PBS containing 1% BSA (5 min, 500 × g, room temperature) and suspended in 1 mL of PBS containing 1% BSA and subjected to FC analysis, using BD FACS Aria II Cell Sorter, Beckton Dickinson and analysed using BD FACS Diva Software v6.1.3.

### Immunofluorescence staining

The immunofluorescence staining was performed in paraffin-embedded endometrial sections (n = 3 per category, ∑n = 12) in order to determine the localization of IFN-γ, IL-4, IL-4R1, IL-13, and IL-13RA. The deparaffinized sections were washed three times with PBS containing 0.1% Tween 20 (Sigma), permeabilized with PBS containing 0.5% TritonX-100, blocked for 1 h in room temperature with fish serum blocking buffer (Thermo Scientific, 377,527), followed by overnight incubation with primary antibodies at 4℃ (IFN-γ, PB028E, KingFisher, 1:50, rabbit; IL-4 KingFisher, PB0052E 1:50, goat; IL-4RA Ab203398, Abcam, 1:100, rabbit; IL-13, KP1557E, KingFisher, 1:50, rabbit; IL-13RA, Ab233528, Abcam, 1:100, rabbit). In the following day, cells were washed with PBS containing 0.1% Tween-20, incubated with secondary antibodies (anti-rabbit-AlexaFluor 594 A210207, Thermo Fisher, 1:200; anti-goat-Alexa Fluor-488, A11055, Thermo Fisher, 1:200). After 1 h at room temperature in the wet chamber, slices were washed three times and mounted in Fluoroshield with DAPI (F6057, Sigma). Negative controls were performed in the absence of primary antibodies and are indluded in Supplementary Fig. 1. Quantitative image analysis was performed in ImageJ on immunostained sections by manually delineating regions of interest for luminal epithelium (LE), glandular epithelium (GE), and stroma; for each ROI mean staining intensity was measured normalized to area after background subtraction. To ensure comparability without whole-slide analysis, analyzable fields were defined to include LE and GE and summarized results are presented in the Supplementary Fig. 2.

### RNA extraction

For qPCR analysis, total RNA from endometrial tissue samples (n = 6 per category ; ∑n = 24) was extracted using mirVana isolation kit (Invitrogen, AM1560) according to the manufacturer’s instructions. For qPCR and NGS analyses, total RNA was extracted from cultured fibroblasts (n = 4) using TRI Reagent according to the manufacturer’s instructions. After treatment, cells were washed with sterile PBS, lysed using TRI Reagent, and total RNA was extracted according to the manufactures instructions. The concentration and quality of total RNA was determined spectrophotometrically. The ratio of absorbance at 260 and 280 nm (A260/280) was approximately 2. RNA integrity number (RIN) was assessed for each RNA isolate using Agilent 2100 system and Expert software (Agilent Technologies, Inc., Santa Clara, CA, USA). All samples had RIN approximately 10 and were processed further.

### Library preparation and next generation sequencing (NGS) of RNA isolated from mare endometrial fibroblasts

Standard library preparation steps including mRNA selection, fragmentation, cDNA synthesis, end repair, adenylation, indexed adapters ligation, and amplification were followed by a qualitative evaluation (Agilent TapeStation 2200) and quantitation (Qubit, Thermo Fisher Scientific). In total, 1000 ng of RNA was used for library construction with the TruSeq RNA Sample Prep v2 kit (Illumina, San Diego, CA). The libraries were sequenced in paired-end 151 bp run (2 × 151 bp) on the NovaSeq 6000 system using the TruSeq Stranded mRNA LT Sample Prep kit, to obtain 15–20 million reads/sample. The NGS analysis was performed by Macrogen Europe, Netherlands**.**

### The bioinformatic analysis of NGS data

RNA-seq data generated as part of this study have been deposited in the Sequence Read Archive (SRA) under accession number PRJNA1152744. The raw reads underwent initial quality assessment and clean-up procedures using FastQC^109^ and Trimmomatic^110^. Subsequently, the reads were aligned to the horse reference genome (EquCab3.0) employing STAR^111^, followed by read counting using feature Counts 2.0.3^112^. To compare transcriptomic profiles across samples, Principal Component Analysis (PCA) was conducted on vst-transformed count values^113^, revealing distinct major clusters corresponding to each category. Differential gene expression analysis was performed using DESeq2^113^, with a predefined criterion of an absolute value of log_2_fold change (log2FC) ≥ 1.0 and a false discovery rate (FDR) adjusted p value (padjusted) < 0.05 to identify significantly differentially expressed genes (DEGs). To identify the biological functions associated with these genes, gene ontology (GO) enrichment analysis was carried out using the R package clusterProfiler^114^ with Fisher’s exact test. Gene names were mapped to GO terms utilizing the org.Hs.eg.db package^115^, and only GO terms with an padjusted < 0.05 were considered significantly enriched. To obtain a more comprehensive view of gene functions and interactions gene enrichment was also performed based on the Kyoto Encyclopedia of Genes and Genomes (KEGG) database^116–118^ using clusterProfiler^119^. Kyoto Encyclopedia of Genes and Genomes categories exhibiting an padjusted < 0.05 were considered significantly enriched. The results of the analysis were visualized using R (R Core Team, 2021).

### qPCR

Total RNA (2 µg) was treated with DNase (AMPD1, Sigma) and subsequently reverse transcribed using a High-Capacity cDNA Reverse Transcription Kit (4,368,814; Invitrogen) according to the manufacturer’s directions. The cDNA was stored at − 20 °C until qPCR. Further, qPCR was performed in a final volume of 10 µL, using 3 µL (10 ng) of cDNA, 0.5 µL of specific primers with probes, 5 µL TaqMan Universal PCR Master Mix II (4,440,049; Applied Biosystems; Thermo Fisher Scientific, Wilmington, NC, USA), and 1.5 µL nuclease-free water, on 384-well plates. All samples were run in duplicates. Amplification was performed with initial denaturation for 10 min at 95 ℃, followed by 45 cycles of 15 s at 95 ℃ and 60 s at 60 ℃ with 7900HT Real-Time PCR System (Agilent Technologies, Waltham, MA, USA). As a negative control, nuclease-free water instead of template cDNA was used.

The qPCR data were analysed by the method described previously^120^. It is a method for quantifying qPCR results using calculations based on the kinetics of individual PCR reactions without the need for the standard curve, independent of any assumptions or subjective judgments, which allows direct calculation of efficiency and CT. The relative concentration of mRNA (R0) for each target and reference RNAs (*SDHA and HPRT*^10^) was calculated using the equation R0 = 1/(1 + E)^Ct^, where, E is the average gene efficiency and Ct is the cycle number at the threshold. The relative mRNA transcription was calculated as R0target gene/R0reference gene and was expressed in arbitrary units. NormFinder^121^ was used to confirm the selection of the most stable reference genes for the normalization of the results.

### Protein extraction

The protein extraction was performed on mare endometrial tissue using RIPA buffer (Cayman 1,001,026 containing 10 mL of 250 mM Tris–HCl, pH 7.6, containing 750 mM sodium chloride, 5% NP-40, 2.5% sodium deoxycholate, and 0.5% SDS) in the presence of a protease inhibitors (S8820, SIGMAFAST™, Sigma) and phosphatase inhibitor (4,906,845,001, PhosSTOP™, Roche). For protein extraction, 100 mg of mare’s frozen endometria (n = 6/category; ∑n = 24) were grinded with liquid nitrogen in a prechilled mortar and pestle sitting in a bed of dry ice. Subsequently, both tissue homogenates and cell lysates were sonicated, incubated on ice for 1 h and centrifuged at 12 000 g for 15 min at 4 °C. The protein concentration was determined using the bicinchoninic acid protein assay kit (BCA1; Sigma). The protein samples were stored at − 80°C for further analysis.

### Western blot

Equal amounts (20 μg) of protein were dissolved in SDS gel-loading buffer (0.25 M Tris–HCl, pH 6.8, 10% SDS, 50% glycerol, and 0.25% bromophenol blue, 2% b-mercaptoethanol), heated to 95 °C for 5 min and separated on a 10% SDS–PAGE according to protein molecular weight. Separated proteins were electroblotted using a semi-dry transfer method onto PVDF membranes (Immobilon-P Transfer Membrane; # IPVH00010; Millipore, Billerica, MA, USA). After blocking in 5% non-fat dry milk in TBS-T buffer (Tris-buffered saline, containing 0.1% Tween-20) for 1 h at room temperature, the membranes were further incubated overnight with primary antibodies at 4 °C (GAPDH Ab8245, Abcam, 1:10,000, IFN-γR1, AF7734, Affinity, 1:1000; IL-4RA Ab203398, Abcam, 1:1000; IL-13RA, Ab233528, Abcam, 1:1000). GAPDH protein abundance was used as an internal control. Negative controls were performed in the absence of primary antibodies. Subsequently, the proteins were detected by incubating membranes for 1.0 h at room temperature with secondary antibodies (GAPDH: anti-mouse-HRP A90-116P, Bethyl, 1:7500; IFN-γR1, IL-4RA, and IL-13RA: anti-rabbit-HRP, Ab205718, Abcam, 1:25,000). After washing again with TBS-T, signals were detected by chemiluminescence using Westar Eta C Ultra 2.0 HRP Substrate (XLS075; Cyanagen, Bologna, Italy). The intensity of the immunological reaction in the tissues was estimated by measuring the optical density in the defined area by computerized densitometry using Image Lab Software version 6.0 (Bio-Rad Laboratories).

### Enzyme-linked immunosorbent assay (ELISA)

The enzyme-linked immunosorbent assay (ELISA) was employed to determine the protein concentration of IFN-γ (EHS0005, Fine Test), IL-4 (EHS0032, Fine test), and IL-13 (orb779722, biorbyt) in the lysates of mare endometrial tissue (n = 6/category; ∑n = 24). The analyses were performed following manufacturer’s instructions. The results were calculated as a pg of IFN-γ/IL-4/IL-13 per 1µg of protein.

### Statistical analysis

All data were presented as mean ± SD. The normal distribution of continuous variables was tested using the Shapiro–Wilk test (GraphPad Prism version 10.2.0 for Windows, GraphPad Software, San Diego, California USA, www.graphpad.comGraphPad). Whenever the assumptions of normal distribution were not met, nonparametric statistical analyses were done. The results were considered significantly different when P < 0.05. GraphPad Prism 10.0 was used both to perform statistical analysis and for generating graphs.

## Supplementary Information

Below is the link to the electronic supplementary material.

Supplementary Material 1

Supplementary Material 2

Supplementary Material 3

Supplementary Material 4

Supplementary Material 5

Supplementary Material 6

## Data Availability

The raw reads have been deposited in the Sequence Read Archive (SRA) under accession number PRJNA1152744.
