# Supplementary material for: Th1 and Th2 cells in equine endometrosis and their interactions with endometrial fibroblasts
Source: Sci Rep. 2025 Oct 16;15:36263. doi: 10.1038/s41598-025-20152-0 (PMC12533255; doi:10.1038/s41598-025-20152-0)
Supplement: Supplementary file 5 — Supplementary Material 5 [file 41598_2025_20152_MOESM5_ESM.docx]

**
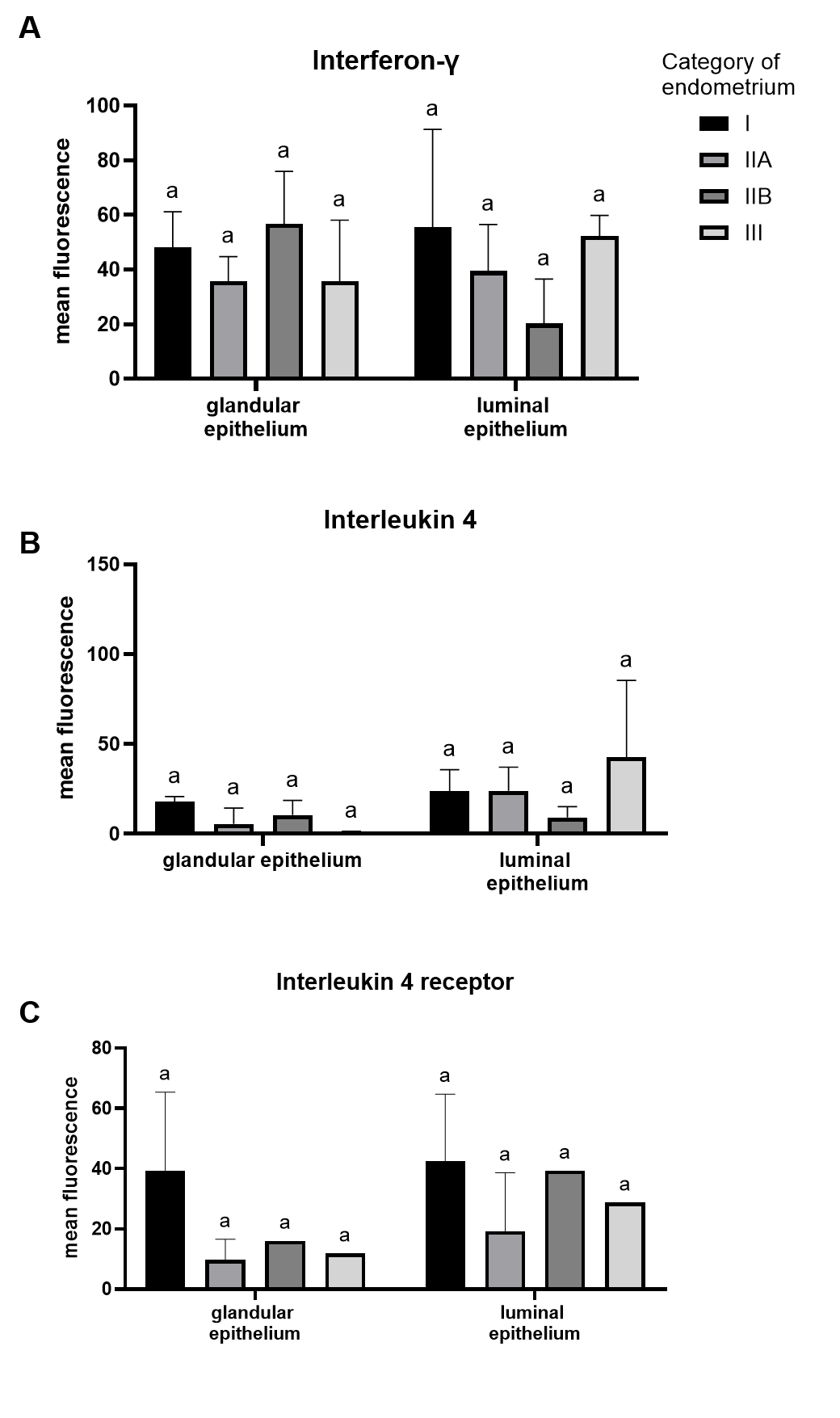
**

**Supplementary Figure 2.** Quantification mean intensity of the interferon γ (A), interleukin 4 (B), and interleukin 4 receptor (C) staining. Bar graphs show ImageJ measurements of immunostaining intensity in luminal epithelium and glandular epithelium for each target across the sampled endometrial sections. Regions of interest were manually delineated (background subtracted). Bars represent mean signal per area (arbitrary units) ± SD; per-slide ROIs were averaged prior to statistics. Results were analysed using two-way ANOVA. While trends in compartmental localization are evident, no significant differences were detected. The intensity of IL-13 and IL-13R immunostaining were not calculated owing to insufficient signal and high background noise.
