# Supplementary material for: Th1 and Th2 cells in equine endometrosis and their interactions with endometrial fibroblasts
Source: Sci Rep. 2025 Oct 16;15:36263. doi: 10.1038/s41598-025-20152-0 (PMC12533255; doi:10.1038/s41598-025-20152-0)
Supplement: Supplementary file 6 — Supplementary Material 6 [file 41598_2025_20152_MOESM6_ESM.docx]

**
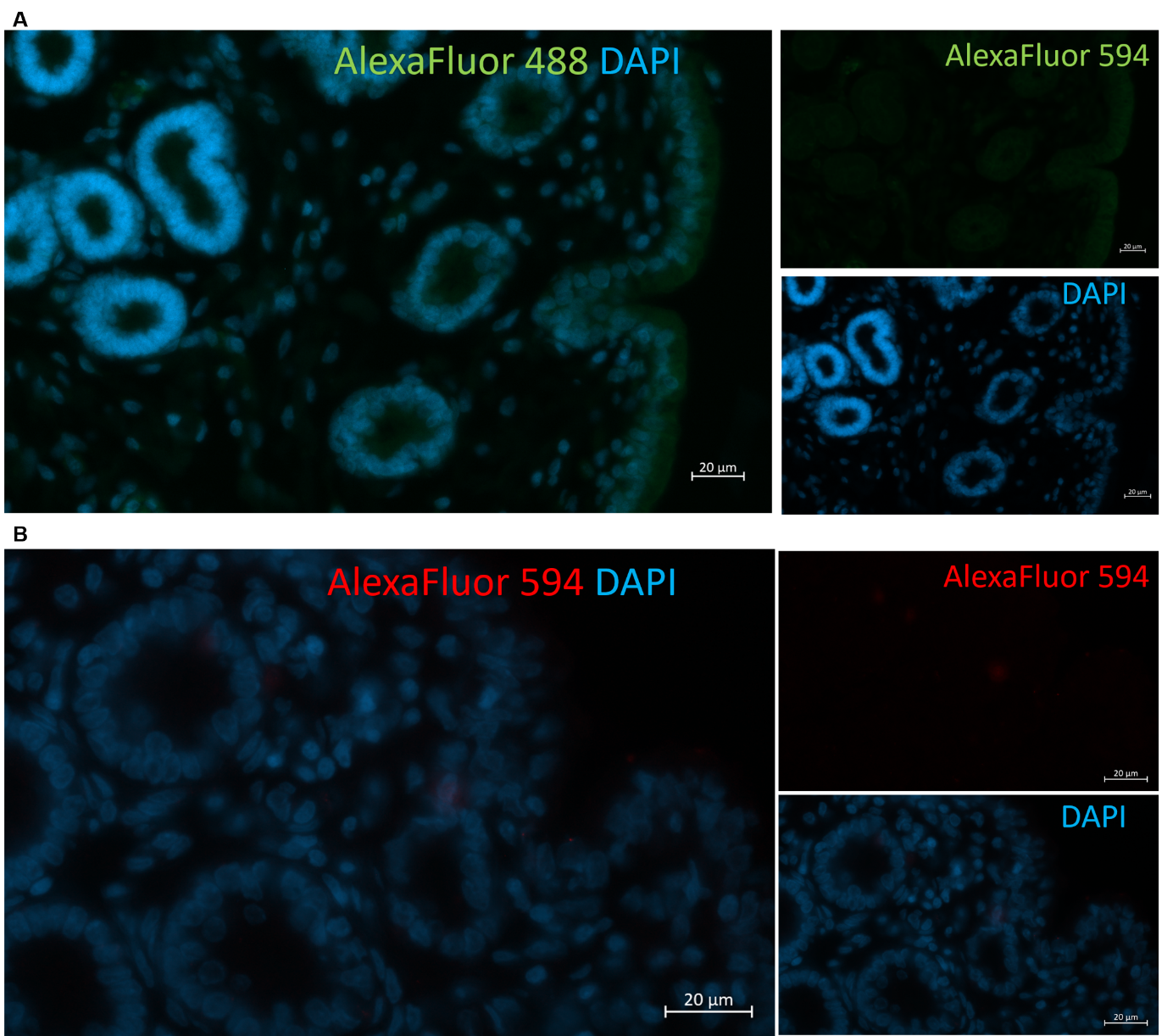
****Supplementary Figure 1.**  The negative control of Alexa Fluor 488 (A) and Alexa Fluor 594-conjugated secondary antibody. Bar = 20 µm.
